# Plant-Derived Phytonutritional Ingredients for Food and Pet Food: Processing, Functionality, and Applications

**DOI:** 10.3390/foods15213859

**Published:** 2026-10-28

**Authors:** Phatthranit Klinmalai, Pitiya Kamonpatana, Atcharawan Srisa, Phanwipa Wongphan, Khwanchat Promhuad, Anusorn Seubsai, Nathdanai Harnkarnsujarit

**Affiliations:** 1 Faculty of Agro-Industry, Chiang Mai University, Samut Sakhon 74000, Thailand; phatthranit.k@cmu.ac.th; 2 Department of Food Science and Technology, Faculty of Agro-Industry, Kasetsart University, Bangkok 10900, Thailand; fagipyk@ku.ac.th; 3 Department of Packaging and Materials Technology, Faculty of Agro-Industry, Kasetsart University, Bangkok 10900, Thailand; atcharawan.sri@ku.th (A.S.); phanwipa.w@ku.th (P.W.); khwanchatpromhuad@gmail.com (K.P.); 4 Department of Chemical Engineering, Faculty of Engineering, Kasetsart University, Bangkok 10900, Thailand; fengasn@ku.ac.th; 5 Center for Advanced Studies for Agriculture and Food (CASAF), Kasetsart University Institute for Advanced Studies (KUIAS), Kasetsart University, 50 Ngam Wong Wan Rd., Latyao, Chatuchak, Bangkok 10900, Thailand

**Keywords:** phytonutritional matrix, plant-derived ingredients, functional food, companion animal nutrition, food processing

## Abstract

Processing modifies the structure, nutritional characteristics, and technological properties of plant-derived food matrices, but findings from isolated ingredients and experimental models do not necessarily describe their performance in finished foods. This selective narrative review examines five processing-responsive matrix categories relevant to functional-food development: legume-derived protein–carbohydrate matrices, resistant and slowly digestible carbohydrates, plant-derived dietary fibers, prebiotic fibers and oligosaccharides, and fermented plant ingredients. Evidence from human-food research is synthesized to examine the effects of thermal, enzymatic, mechanical, pressure-based, extraction, and fermentation processes on matrix properties, digestibility, fermentability, and food applications. Physicochemical, in vitro, cellular, animal-model, and human-intervention findings are distinguished according to their experimental systems. Feeding studies in dogs and cats are considered separately as application-level evidence from formulated diets, with emphasis on nutrient digestibility, fecal characteristics, fermentation metabolites, microbiota responses, postprandial glycemic responses, and dietary acceptance. Reported outcomes differ with ingredient composition, processing conditions, inclusion level, and dietary formulation, while the heterogeneity and limited duration of many feeding studies restrict conclusions about longer-term physiological effects. Across the selected literature, matrix properties measured before food manufacture require separate evaluation after formulation, and experimental indicators of functionality should not be treated as demonstrated health benefits without appropriate evidence. The review provides a processing–matrix–function framework for interpreting plant-derived ingredients in finished foods while identifying limitations in the assessment of their physiological relevance.

## 1. Introduction

Plant-derived ingredients have become important components of functional food development because they contain proteins, starches, dietary fibers, oligosaccharides, and phytochemicals within structurally complex food matrices. Their functionality is influenced not only by chemical composition but also by the physical organization and accessibility of these constituents during processing and digestion. Enzymatic hydrolysis, thermal treatment, pressure processing, particle-size reduction, extraction, and fermentation can modify protein hydrolysis, starch organization, fiber architecture, and the availability of bound compounds [1,2]. Understanding these changes is important for designing plant ingredients with defined nutritional and technological properties. Legumes provide a useful example because protein, starch, fiber, and phytochemicals coexist within the same cellular matrix. Processing can generate bioactive peptides, alter protein digestibility, modify starch accessibility, and change antioxidant properties [3,4]. Plant starches show comparable sensitivity to processing. Amylose content, molecular interactions, thermal history, and mechanical treatment influence the proportions of rapidly digestible, slowly digestible, and resistant starch, with consequences for digestion kinetics [1,5,6]. Dietary fibers are similarly responsive to extraction and modification, which can alter particle structure, porosity, hydration, adsorption capacity, and the accessibility of fiber-associated phenolics [7,8,9]. This structural perspective also extends to prebiotic and fermented plant ingredients. Oligosaccharides, β-glucans, pectin, and inulin differ in digestive resistance and microbial utilization according to molecular size, linkage pattern, extraction conditions, and their incorporation into food matrices [10,11,12,13]. Fermentation introduces an additional route for modifying plant materials through microbial metabolism and enzymatic activity, with reported changes in antinutritional compounds, proteins, phenolics, polysaccharides, and fermentation-derived metabolites [2,14]. In this review, functionality refers to the measurable technological and nutritional properties of plant-derived food matrices. Technological functionality includes hydration, swelling, viscosity, gelation, and emulsification, whereas nutritional properties include nutrient accessibility and digestibility under the conditions evaluated. These properties must be distinguished from those of the finished food, where processing and interactions among ingredients may affect their expression. Functional foods are finished products intended to provide physiological benefits in addition to basic nutrition. Such benefits cannot be established from physicochemical characterization or in vitro findings alone and require evidence from appropriate in vivo studies.

Commercial applications of plant-derived ingredients have expanded across food formulations, including alternative proteins, dietary fibers, and plant-based products. In companion-animal foods, commercial developments include plant-based and hybrid formulations alongside conventional diets. Product-label analyses have identified differences in nutrient composition, pricing, and marketing claims among these categories [15]. Recent market analyses also document substantial pet food product-launch activity, although these data do not establish the market share of individual plant-derived ingredients [16]. These developments emphasize the importance of evaluating ingredient functionality within commercially relevant food formulations. Health-related claims also require consideration beyond experimental measurements of ingredient functionality. Within the European Union, nutrition and health claims for foods intended for human consumption are governed by Regulation (EC) No 1924/2006, which requires appropriate scientific substantiation for authorized health claims [17]. Pet food labelling and associated claims are subject to a separate framework under Regulation (EC) No 767/2009 [15]. Accordingly, physicochemical properties, in vitro activity, and isolated feeding-trial responses should not be treated as sufficient evidence for product-level health claims without appropriate substantiation.

Existing reviews have addressed plant-derived functionality from several distinct perspectives. Bioactive-centered reviews have focused on phytochemicals, extraction, and reported human-health effects [18,19], while protein-focused reviews emphasize nutritional quality, technological functionality, and sustainable food applications [20,21]. Other reviews examine fermented plant foods [22] or prebiotic plant polysaccharides and gut microbiota [23]. Food-matrix effects on the bioaccessibility of plant bioactive and micronutrients have also received recent attention [24]. These areas, however, are generally addressed as separate ingredients or functional categories. Less attention has been given to how food processing modifies multiple classes of plant-derived nutritional matrices within a common framework and how these properties are expressed after incorporation into finished foods. Our previous review examined phytochemical-rich ingredients and other plant-derived bioactives in companion-animal nutrition, with emphasis on their classification, reported biological activities, and feeding applications [25]. The present review addresses a different scientific question by focusing on processing-responsive food matrices, including legume-derived protein–carbohydrate systems, resistant and slowly digestible carbohydrates, dietary fibers, prebiotic fibers and oligosaccharides, and fermented plant ingredients. Four original studies are cited in both reviews [26,27,28,29]. These studies are considered here in relation to processing, food formulation, and measured responses after dietary incorporation. The present review is organized around processing–matrix–function relationships, with human-food research providing the primary context for examining nutritional and technological properties.

Companion-animal feeding studies are considered as a separate application context in which plant-derived ingredients are incorporated into formulated diets for dogs and cats. Their reported responses provide evidence of nutritional and physiological outcomes under the tested feeding conditions but do not necessarily establish whether specific structural properties measured before manufacture persist in the finished food. These findings are not used to predict physiological outcomes in humans. In this review, a phytonutritional matrix refers to a plant-derived ingredient or ingredient system containing proteins, carbohydrates, dietary fibers, oligosaccharides, and associated constituents whose organization and accessibility may be modified during processing. The five matrix categories are used to organize the evidence according to the principal ingredient characteristics, processing intervention, and experimental question; they are not mutually exclusive classes of plant materials. The aim of this review is to examine processing-related changes in plant-derived matrices, distinguish their experimentally measured technological and nutritional properties from physiological responses, and assess their relevance after incorporation into formulated foods. Physicochemical, in vitro, cellular, animal-model, and human-intervention findings are interpreted according to their respective experimental systems, without treating isolated ingredient properties as demonstrated physiological benefits.

## 2. Review Methodology

This review was conducted as a selective narrative review using a targeted literature search in Scopus. Searches were performed on 30 July, 2026, using title, abstract, and keyword fields (TITLE-ABS-KEY) across five predefined matrix domains: legume-derived protein–carbohydrate matrices, resistant and slowly digestible carbohydrates, plant-derived dietary fibers, prebiotic fibers and oligosaccharides, and fermented plant ingredients. For human-food research, matrix-specific terms were combined with “functional food”. The search vocabulary covered plant materials, matrix components, and processing approaches, including dietary fiber, cellulose, hemicellulose, pectin, bran, resistant starch, starch digestibility, glycemic response, extrusion, gelatinization, retrogradation, inulin, fructooligosaccharides, galactooligosaccharides, xylooligosaccharides, β-glucan, legume proteins, and fermented plant ingredients. Searches were restricted to original research articles. The selected literature included studies relevant to human-food development, with publication-year coverage considered separately from the filters applied in individual searches.

Companion-animal literature was examined separately using matrix-specific terms combined with dog, canine, cat, feline, pet food, or companion animal. Studies published between 2016 and July 2026 were considered to accommodate the more limited literature on plant-derived ingredients in formulated dog and cat diets. The term “functional food” was not required for these searches because companion-animal feeding studies are not necessarily indexed using this designation. Scopus records were ranked by relevance, and screening was restricted to the first 20 records for each matrix-specific search. This restriction may have excluded relevant publications appearing later in the ranking. Accordingly, the review was intended to provide a focused qualitative synthesis and does not claim comprehensive coverage of either literature set.

Articles were selected according to their relevance to the processing–matrix–function framework. Eligible studies examined plant-derived ingredients or food matrices and reported findings concerning composition, processing, formulation, physicochemical properties, technological performance, digestibility, fermentation, microbiota responses, metabolic measurements, or sensory characteristics. Companion-animal studies were included when plant-derived ingredients were evaluated in diets administered to dogs or cats and nutritional, physiological, gastrointestinal, or feeding-related outcomes were measured. Reviews, non-original research publications, non-plant interventions, and studies without a direct relationship to the review scope were excluded. Titles and abstracts were screened for relevance, and full texts were examined to determine experimental conditions, measured outcomes, and suitability for inclusion. Duplicate publications appearing in multiple matrix searches were retained only once in the study inventory.

Studies were assigned to one primary matrix category according to the principal intervention and experimental question. Investigations centered on microbial fermentation during ingredient preparation were classified as fermented plant ingredients; studies examining microbial utilization of selected carbohydrates as prebiotic fibers and oligosaccharides; investigations of starch structure and digestion kinetics as resistant and slowly digestible carbohydrates; studies emphasizing fiber structure and physicochemical properties as dietary fibers; and studies examining integrated protein–carbohydrate properties of legume ingredients as legume-derived matrices. The categories were used to organize the evidence and were not treated as mutually exclusive ingredient classes.

**Figure 1:**
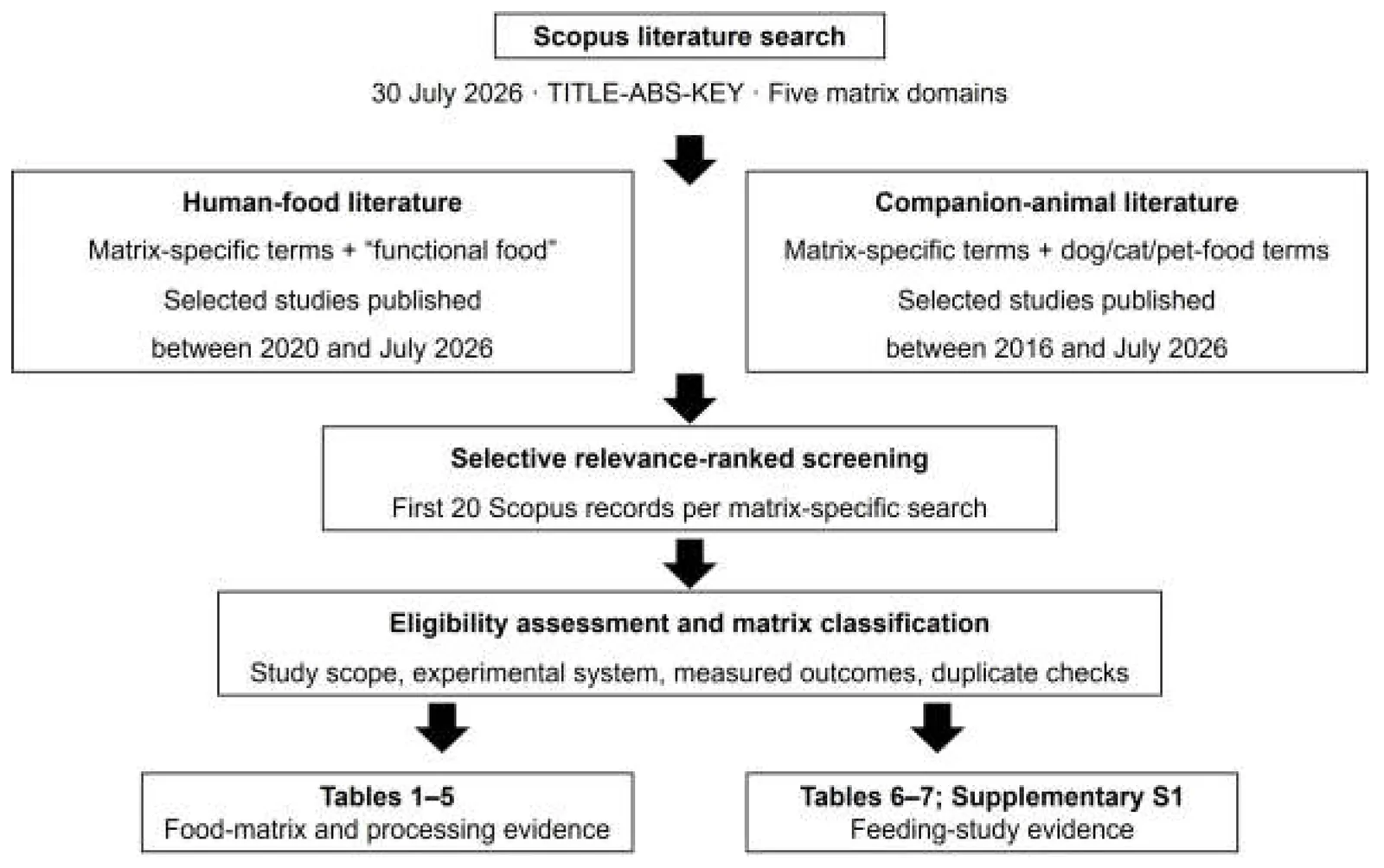
Overview of the selective literature search and evidence classification.

For Table 1, Table 2, Table 3, Table 4 and Table 5, the synthesis considered raw materials, processing and incorporation conditions, food applications, experimental systems, measured endpoints, and reported findings. Physicochemical, in vitro, cellular, animal-model, and human-intervention evidence were distinguished according to the experimental methods employed. Companion-animal feeding evidence was synthesized separately in Table 6 and Table 7, with attention to processing and formulation context, measured outcome domains, and reported responses. Study-level information on species, sample size, experimental design, feeding duration, ingredient inclusion level, comparator, and direction of reported effects was compiled in Supplementary Table S1. Figure 1 summarizes the literature-selection and evidence-classification approach.

The synthesis was qualitative and did not involve meta-analysis or formal risk-of-bias assessment. Interpretation considered differences in processing conditions, experimental systems, dietary formulations, comparators, feeding durations, and measured endpoints. Changes in nutrient digestibility, fecal metabolites, or microbiota composition were not interpreted as evidence of clinical benefits without corresponding physiological or clinical measurements. Conclusions were limited to the selected studies, recognizing the potential selection bias associated with relevance-ranked screening and the heterogeneity of the experimental evidence.

## 3. Plant-Derived Functional Matrices: Processing, Functionality, and Food Applications

### 3.1. Legume-Derived Protein–Carbohydrate Matrices

Legume-derived protein–carbohydrate matrices provide examples of how processing affects nutritional and technological properties at different levels of food organization (Table 1). Enzymatic treatment of chickpea, lentil, and other legume proteins yielded different peptide profiles and in vitro antioxidant or ACE-inhibitory activities, depending on protein source, enzyme conditions, and fractionation [3,30,31]. These assay results do not establish corresponding physiological effects following consumption. Heat treatment also affected the hydrolysis and peptide profiles of isolated legume proteins during simulated digestion [32], while high-pressure homogenization altered pea starch–polyphenol complexes and their in vitro enzymatic digestibility under different pH conditions [33]. Milling, fractionation, annealing, and pressure heating yielded further differences in starch crystallinity, hydration, rheological behavior, and digestible starch fractions [34,35,36,37]. Studies using formulated foods address a distinct level of functionality. Legume-containing tarhana, composite porridge, and lentil noodles exhibited changes in nutrient composition and product properties, including viscosity, hydration, texture, and sensory acceptability [38,39,40]. The glycemic index reported for lentil-enriched noodles was predicted using an in vitro procedure and should not be interpreted as a human postprandial response [40]. Likewise, pea-enriched bread showed formulation-related differences in hardness and simulated digestion under adult and elderly gastrointestinal conditions [41]. Chickpea-derived proteins, polysaccharides, and oligosaccharides produced microbial and fermentation-related responses in experimental models [3,42,43]; these observations indicate substrate utilization under the conditions tested but do not establish prebiotic effects in consumers. Overall, the studies show that processing-related properties measured in isolated ingredients and model systems require evaluation in formulated foods before their product-level relevance can be determined. Companion-animal feeding studies involving legume-derived ingredients are discussed in Section 4 as application-level evidence from complete diets. Their measured outcomes should not be taken as direct evidence that particular structural features found in isolated ingredients persist after food manufacture.

**Table 1:** Legume-derived protein–carbohydrate matrices in functional foods and representative evidence supporting their processing-related functionality.

Raw Materials	Processing/Incorporation Approach and Level	Food Matrix/Application Form	Experimental System	Measured Endpoints	Reported Findings	Ref.
Chickpea albumin and globulin proteins	Protein extraction followed by enzymatic hydrolysis	Functional peptide ingredient	In vitro fecal fermentation; antioxidant assay	– Microbiota composition (beneficial bacterial genera) – Fermentation metabolites (short-chain fatty acids, ammonia, indole) – Antioxidant activity (radical scavenging capacity)	In an in vitro fecal fermentation model, chickpea albumin-derived peptides showed the highest antioxidant assay activity among tested fractions, with higher abundance of selected bacterial groups and SCFA production and lower ammonia and indole formation.	[3]
Chickpea and lentil protein	Enzymatic hydrolysis using bromelain, ficin, and papain at controlled concentrations (2.5–10%) and durations (1, 2, and 12 h)	Protein hydrolysates	Protein hydrolysis; physicochemical characterization	– Degree of hydrolysis (protein breakdown efficiency) – Peptide profile (molecular weight distribution) – Amino acid composition (free amino acid release) – Protein structural changes (SDS-PAGE analysis)	Papain produced the highest degree of hydrolysis under the conditions tested (34.8% for lentil protein at 10% enzyme, 12 h); enzyme type, concentration, and duration affected peptide-size profiles and free amino acid release.	[30]
Lentil, black soybean, black turtle bean proteins	Enzymatic hydrolysis followed by ultrafiltration (<3 kDa) and chromatographic fractionation	Bioactive peptide fractions	In vitro enzyme-inhibition and antioxidant assays	– Enzyme inhibition (ACE inhibitory activity, IC50) – Antioxidant activity (multiple radical-scavenging assays) – Peptide characterization (molecular weight distribution, sequence analysis) – Protein digestibility and hydrolysis	Peptide fractions below 3 kDa showed greater ACE inhibition in vitro; antioxidant assay responses varied with fraction and legume source and did not uniformly increase with decreasing molecular size.	[31]
Chickpea polysaccharides	Ultrasound-assisted extraction combined with freeze–thaw treatment	Isolated polysaccharide	Extraction; simulated digestion; in vitro fecal fermentation	– Extraction efficiency (yield) – Antioxidant activity (DPPH, ABTS assays) – In vitro digestion (stability and degradation) – Fermentation metabolites (short-chain fatty acids) – Microbial response (probiotic growth)	Ultrasound–freeze–thaw extraction yielded water-soluble chickpea polysaccharides with radical-scavenging activity; simulated digestion showed partial degradation and in vitro fecal fermentation showed SCFA production and selected bacterial responses.	[42]
Himalayan green pea and green chickpea	Autoclaving, roasting, boiling, and sonication	Processed Himalayan green pea and green chickpea ingredients	Processed ingredient characterization; in vitro digestibility	– Nutritional composition (protein content, dietary fiber levels) – Protein digestibility (in vitro digestibility assessment) – Phenolic profile (catechin, rutin, gallic acid, ferulic acid) – Antioxidant capacity (bioactive compound analysis)	Autoclaving, roasting, boiling, and sonication changed composition, phenolic profiles, and in vitro protein digestibility of Himalayan green pea and green chickpea; reported 23–26% values refer to increases in protein content, not final protein concentration.	[44]
Black soybean, mung bean, white kidney bean, and faba bean proteins	Heat treatment (95 °C, 15 min) followed by in vitro gastrointestinal digestion	Isolated protein systems	Heat-treated isolated proteins; in vitro digestibility	– Protein digestibility (degree of hydrolysis) – Peptide profile (molecular weight distribution, bioactive peptides) – Amino acid release (free amino acids) – Protein structure (secondary structure composition)	Following heat treatment and simulated gastrointestinal digestion, faba bean protein had the highest degree of hydrolysis among four tested proteins, whereas black soybean protein had the lowest; mung bean digestion products contained abundant low-molecular-weight peptides and free amino acids.	[32]
Pea protein concentrate	Partial substitution of wheat flour with untreated or high-pressure homogenization treated 5% pea protein during bread formulation	Pea protein-enriched bread	Formulated bread; in vitro protein digestibility and colonic fermentation	– Protein digestibility (in vitro gastrointestinal digestion models) – Colonic fermentation (microbial fermentation potential, metabolite formation) – Physical properties (bread hardness, structural characteristics)	In pea-protein-enriched bread, high-pressure homogenization and reformulation affected bread hardness, simulated protein digestion, and subsequent in vitro colonic fermentation; digestibility was lower under simulated elderly than adult conditions.	[41]
Pulse flours (pinto bean, chickpea, yellow pea, red lentil)	Substitution of at 50% wheat flour with pulse flours during tarhana formulation and fermentation	Tarhana food product	Formulated fermented food; physicochemical and in vitro digestibility	– Nutritional composition (protein content, mineral content) – Protein digestibility (in vitro digestibility) – Functional properties (rheological and structural characteristics) – Phenolic and antioxidant properties	Replacing 50% of wheat flour with selected legume flours increased protein, mineral, and phenolic content of tarhana; in vitro protein digestibility remained above 78%, while soup viscosity was lower in substituted formulations.	[38]
Composite flour from proso millet, little millet, and soaked faba bean	Composite flour formulation with soaked and germinated faba bean incorporated into millet-based mix	Gluten-free ready-to-mix porridge	Formulated porridge; physicochemical and in vitro digestibility	– Nutritional composition (protein, carbohydrate content) – Functional properties (water absorption index, water solubility index) – Pasting properties (gelatinization behavior) – In vitro starch digestibility – Microstructure analysis	Composite millet–faba bean porridges differed in water absorption and solubility indices and pasting behavior; Little millet, proso millet and soaked faba bean formulation had higher in vitro starch digestibility (67.70%) than the other formulations tested.	[39]
Lentil flour	– Lentil flour inclusion levels: 10%, 20%, 30%, and 40% – Blended with wheat flour prior to dough preparation and noodle processing	Lentil-enriched wheat noodles	Formulated noodles; in vitro protein digestibility and in vitro predicted glycemic index; product tests	– Nutritional composition (protein, fiber, minerals) – In vitro predicted glycemic index – Functional properties (protein digestibility) – Sensory attributes (texture, flavor, acceptability) – Storage stability (nutritional and sensory retention)	At 30% lentil-flour inclusion, noodles had higher measured protein and fiber contents than control noodles and a predicted glycemic index of 60.57 ± 0.07 derived from an in vitro digestion method.	[40]
Pulse flours and starches (pigeon pea, cowpea, pinto bean, navy bean)	Fractionation into flour vs. starch fractions	Functional pulse-derived flour and starch ingredients	Isolated flours and starches; physicochemical and in vitro digestibility	– Functional properties (water absorption capacity, oil absorption capacity, swelling power, solubility) – Structural properties (crystallinity, microstructure) – Rheological properties (flow behavior, viscosity) – Starch digestibility (rapidly digestible starch, slowly digestible starch, resistant starch)	Pinto bean flour and starch had the highest water absorption capacity, and cowpea flour and starch the highest oil absorption capacity, among the materials tested; flour and starch fractions differed in rheology and in vitro starch fractions.	[36]
Pea starch–polyphenol complexes (gallic acid, ferulic acid, quercetin, tannic acid)	High-pressure homogenization to form starch–polyphenol complexes under controlled pH conditions (5, 7, and 9)	Model pea starch–polyphenol system	Model starch–polyphenol complexes; in vitro digestibility	– Starch digestibility (rapidly digestible starch, slowly digestible starch, resistant starch) – Structural properties (crystallinity, molecular interactions) – Thermal and rheological properties – Complex formation index	At lower pH, high-pressure-homogenized pea starch–polyphenol complexes exhibited increased complex formation and enhanced crystalline ordering, as well as reduced enzymatic digestibility in vitro; responses depended on polyphenol identity.	[33]
Pea starch fractions (fine starch, coarse starch, parent starch)	Milling and air classification to isolate starch fractions with different particle size distributions	Isolated starch fractions	Isolated starch fractions; physicochemical and in vitro digestibility	– Particle size distribution – Structural properties (granule morphology, molecular order) – Thermal properties (enthalpy changes) – Pasting properties (viscosity profiles) – Starch digestibility (fast-digesting starch, resistant starch)	Fine starch obtained by milling and air classification contained less fast-digesting starch and more resistant starch than the compared fractions in vitro; structural, thermal, and pasting properties differed across fractions.	[37]
Pea starch (wet-ground and dry-ground starch fractions)	Comparison of wet grinding and dry grinding processes	Isolated starch ingredients	Isolated starch fractions; physicochemical and in vitro digestibility	– Structural properties (crystallinity, amylopectin chain distribution) – Physicochemical properties (gel hardness, swelling power, viscosity) – Thermal properties (gelatinization temperature) – Starch digestibility (resistant starch content)	Wet-ground pea starch had higher crystallinity, gelatinization viscosity, and swelling power, whereas dry-ground starch had higher resistant starch content and gel hardness under the tested analytical conditions.	[34]
Pea type III resistant starch (RS3)	Produced via acid hydrolysis, pullulanase debranching, followed by annealing (90 °C) and pressure heating (121 °C) treatments	Functional starch ingredient	Modified isolated starch; physicochemical and in vitro digestibility	– Starch digestibility (rapidly digestible starch, resistant starch content) – Structural properties (crystallinity, particle size, gelatinization temperature) – Functional properties (swelling power, pasting viscosity)	Annealing and pressure heating increased resistant starch content and crystalline order while reducing swelling power, pasting viscosity, and rapidly digestible starch; pressure heating produced the highest reported resistant starch content (85.6%) under the tested conditions.	[35]
Resistant starch derived from faba bean	Processed starch incorporated into diet	Experimental diet for diabetic Wistar rat model	Diabetic Wistar rat feeding experiment	– Glycemic markers (blood glucose levels, metabolic indicators) – Biochemical parameters (serum metabolic markers) – Physiological responses (body weight, metabolic status)	In diabetic Wistar rats, dietary replacement with faba-bean resistant starch was associated with lower measured postprandial and fasting glucose indicators than in the untreated diabetic comparison group.	[45]
Oat β-glucan combined with pea starch	Oat β-glucan inclusion levels: 0%, 5%, 10%, 15%, and 20% (*w*/*w* relative to pea starch) in aqueous system	Model starch–polysaccharide system	Model starch–*β*-glucan gel; physicochemical and in vitro digestibility	– Structural properties (particle size, microstructure) – Pasting properties (peak viscosity, final viscosity, pasting temperature) – Starch digestibility (in vitro digestibility) – Molecular interactions (amylose behavior)	Increasing oat *β*-glucan content modified pea-starch pasting and molecular behavior, reduced amylose leaching and short-term retrogradation, and reduced starch digestibility in vitro.	[46]
Chickpea-derived crude oligosaccharides (raffinose family oligosaccharides)	Extracted using 50% ethanol (1:5 *w*/*v*, 60 min) followed by in vitro fermentation and yogurt formulation 2–10% supplementation levels	In vitro simulated gut microbiota fermentation followed by cashew nut yogurt-like product formulation	Simulated fecal fermentation; formulated yogurt-like food	– Microbiota composition (relative abundance of taxa) – Fermentation metabolites (short-chain fatty acids) – Physicochemical properties (pH, stability) – Microbial viability (lactic acid bacteria, *Bifidobacterium* counts)	In simulated fecal fermentation, chickpea oligosaccharides altered selected bacterial taxa and increased SCFA production, particularly with the Kabuli extract. In cashew nut yogurt-like products, tested additions maintained measured physicochemical properties and bacterial viability during refrigerated storage.	[43]

Note: The reported findings cover the experimental systems described. Measurements of structure, technological performance, in vitro digestion, microbial use, and biochemical activity do not show any proven physiological benefits for humans. Testing a formulated food alone does not count as a human intervention. The rat experiment [45] is identified separately.

### 3.2. Resistant Starch and Slowly Digestible Plant Carbohydrates

Resistant and slowly digestible carbohydrate matrices provide a clear example of how processing affects technological properties and starch fractions without necessarily establishing a physiological effect (Table 2). In extruded corn flours, temperature, moisture, and oil incorporation were associated with differences in rapidly digestible, slowly digestible, and resistant starch fractions [1]. Amylose content also affected resistant starch content and 3D-printing performance, with the best dimensional accuracy in the tested starches occurring at an intermediate high-amylose level; subsequent gelatinization and printing reduced resistant starch [47]. These findings indicate that starch composition alone does not describe performance after manufacture. Physical treatments of cassava and rice starch changed molecular organization, retrogradation, hydration, and in vitro digestion profiles [48,49,50]. The effects of added non-starch constituents were less uniform. Highland barley β-glucan increased viscosity and gel hardness while changing starch ordering and resistant starch content [51]. Chia seed gum was found to reduce retrogradation in potato starch but increase it in corn starch, even though both systems exhibited lower in vitro digestibility [52]. Zucchini polysaccharide and kiwifruit fibers also altered starch digestion profiles and pasting behavior in model systems [6,53]. Starch–polyphenol interactions differed between corn and potato starch, with reduced in vitro digestibility and glucose transport measured in a Caco-2 cell model [5]. These findings do not establish lower postprandial glycemic responses in consumers. Studies of composite flours and formulated foods offer a separate level of evidence. Processing of sorghum flours and microwave-treated sorghum meal altered resistant starch fractions, phenolic measurements, gel characteristics, and in vitro hydrolysis [54,55]. Incorporation of unripe pawpaw flour into wheat flour and molokhia leaf powder into flatbread changed starch fractions alongside technological or sensory properties [56,57]. Cassava starch was also used to develop gluten-free noodles, allowing retrogradation-related structural changes to be considered alongside product texture [48]. In contrast to these physicochemical and in vitro investigations, a randomized crossover study in healthy young women measured postprandial responses to oat-based pastes prepared at different roasting temperatures [58]. Under the study conditions, the lower-temperature preparations were associated with lower postprandial glucose responses than the high-temperature and refined-rice comparisons. The human findings are specific to these prepared foods and participants and should not be generalized to other starch matrices. Across these studies, processing changed starch digestibility and technological properties in ways that depended on starch source, non-starch constituents, and formulation. Resistant starch content and estimated glycemic indices remain useful experimental endpoints, but neither independently establishes a physiological benefit from a finished food. Section 4 discusses application-level evidence from complete companion-animal diets, where feeding responses are considered separately from the physicochemical properties measured in ingredient and model systems.

**Table 2:** Resistant starch and slowly digestible plant carbohydrate matrices in functional foods and representative evidence supporting processing-related functionality.

Raw Materials	Processing/Incorporation Approach and Level	Food Matrix/Application Form	Experimental System	Measured Endpoints	Reported Findings	Ref.
Corn flour	Twin-screw extrusion processing at 121, 145, and 160 °C; moisture content at 25%, 30%, and 35%	Ready-to-eat corn flour	Processed corn flour; physicochemical characterization and in vitro digestibility	– Starch digestibility fractions (rapidly digestible starch, slowly digestible starch, resistant starch) – Structural properties (crystallinity, gelatinization behavior) – Processing parameters (temperature, moisture effects)	Processing temperature, moisture and added oil affected the proportions of rapidly, slowly digestible and resistant starch measured in vitro; oil inclusion further altered digestion behavior.	[1]
Corn starch	Corn starches with different intrinsic amylose levels at 28.68%, 58.84%, 66.13%, and 70.06%	3D-printable starch gel system	3D-printed starch gels; rheology, printability and in vitro digestibility	– Starch digestibility (resistant starch content) – Rheological properties (storage modulus, viscosity, shear-thinning behavior) – 3D printability (dimensional accuracy, extrusion behavior) – Structural properties (crystallinity, retrogradation behavior)	At 58.84% amylose, starch showed 33.93% resistant starch and a 6.59% printing-length deviation; higher amylose levels of 66.13% and 70.06% showed poorer printability. Gelatinization and printing also reduced resistant starch.	[47]
Corn starch– and potato starch–polyphenol complexes	Polyphenols complexed (index 2.8–8.3%) with starch matrices through molecular interaction (inclusion and non-inclusion complex formation)	Starch–polyphenol model system	Model starch–polyphenol complexes; in vitro digestion and Caco-2 cell assay	– Starch digestibility (rapidly digestible, slowly digestible, resistant fractions) – Glucose transport (Caco-2 permeability, transporter expression) – Structural interactions (complex formation, molecular structure)	Corn and potato starch formed different polyphenol complexes. Reduced starch digestibility was accompanied by lower glucose transport in Caco-2 monolayers; these are model and cell-based findings.	[5]
Cassava starch	Mechanical activation followed by controlled retrogradation short-term and long-term storage conditions	Gluten-free starch dough and noodles	Formulated gluten-free noodles; physicochemical, texture and sensory evaluation	– Resistant starch content – Structural properties (X-ray diffraction, microstructure, molecular reorganization) – Water mobility (low-field nuclear magnetic resonance) – Texture properties (hardness, elasticity) – Sensory evaluation (color, texture, acceptability)	Longer retrogradation increased resistant starch in mechanically activated cassava starch dough; enhanced starch recrystallization and water redistribution. Noodles prepared from the dough showed different viscoelastic and sensory properties relative to the tested comparator.	[48]
Cassava starch	Physical modification using alternating magnetic field processing (10, 30, and 50 min)	Modified starch system	Modified starch; physicochemical characterization and in vitro digestibility	– Starch structure (molecular order, crystallinity, chain structure) – Physicochemical properties (solubility, swelling power) – In vitro digestibility (starch fraction distribution)	Alternating magnetic-field treatment changed molecular order, solubility and swelling and increased the slowly digestible starch fraction under the tested conditions; the 10 min treatment showed pronounced responses.	[50]
Dual-modified rice starch	Ultrasound-assisted hydrothermal treatment under temperature-controlled conditions	Modified starch system	Modified starch; physicochemical characterization and in vitro digestibility	– Starch digestibility fractions (rapidly digestible starch, slowly digestible starch, resistant starch) – Structural properties (crystallinity, short-range order, particle size) – Thermal properties (gelatinization temperature, enthalpy) – Rheological properties (viscosity, breakdown, viscoelasticity)	Ultrasound-assisted, temperature-controlled hydrothermal treatment changed gelatinization and retrogradation behavior and yielded up to 42.26% slowly digestible starch in the tested rice starch.	[49]
Roasted oat flour (oat-based paste rich in *β*-glucan)	Roasting temperature: 90–110 °C (low temperature) and 160 °C (high temperature)	Oat-based paste	Formulated oat paste; randomized human crossover intervention (21 healthy young female adults), product testing	– Postprandial metabolic response (glucose, insulin, HOMA-PP) – Starch fractions (slowly digestible starch, resistant starch) – Food safety markers (acrylamide content) – Texture and dysphagia compliance	In a 21-participant randomized crossover study, low-temperature roasted oat pastes elicited lower postprandial glucose responses than high-temperature roasted paste and refined-rice reference. Roasting conditions also differed in starch fractions, texture and acrylamide.	[58]
Highland barley β-glucan	Highland barley β-glucan at 1%, 3%, 6%, 9%, and 12% (*w*/*w*)	Starch–β-glucan gel system	Model starch–β-glucan gels; physicochemical and in vitro digestion	– Starch digestibility (resistant starch content) – Rheological properties (viscosity, gel hardness) – Structural properties (crystallinity, molecular order) – Retrogradation behavior (short-term and long-term storage)	Highland barley β-glucan altered gel viscosity, hardness, retrogradation and resistant starch content in a concentration-dependent model system. At 12% addition, resistant starch increased from 19.8% to 25.2–39.3%.	[51]
Chia seed gum	Mixed 5% (*w*/*w*) with potato starch and corn starch prior to gelatinization	Starch–polysaccharide composite gel system	Model starch–β-glucan gels; physicochemical and in vitro digestion	– Starch digestibility (in vitro digestion rate) – Retrogradation properties (enthalpy, peak temperature) – Enzyme activity (α-amylase inhibition) – Structural properties (molecular ordering, gel structure)	At 5% chia gum, retrogradation enthalpy decreased in potato starch (11.59 to 8.49 J/g) but increased in corn starch (4.11 to 5.55 J/g). In vitro digestibility decreased in both models (approximately 75.0 to 70.3% and 74.5 to 69.2%, respectively).	[52]
Red and white sorghum flour	Dry-heat (100 °C, 1 h), moisture (110 °C, 6 h), extrusion at high-temperature short-time processing	Processed sorghum flour	Processed sorghum flours; physicochemical and in vitro digestibility assays	– Starch digestibility (resistant starch content) – Phenolic composition (including flavonoids) – Antioxidant activity (radical scavenging capacity) – Structural properties (granule integrity, starch–lipid complex formation) – Rheological properties (gelation behavior)	Dry heating and extrusion increased resistant starch in the tested sorghum flours; moist heating and extrusion increased antioxidant assay responses. Extrusion reduced measured total phenolics (42.75% in white and 23.06% in red flour). Gel or rheological properties differed between flour types and treatments.	[54]
Sorghum meal with non-tannin and tannin phenolic extracts	Microwave-assisted moisture treatment; phenolic addition	Modified sorghum meal system	Modified sorghum meal; physicochemical and in vitro digestion assays	– Starch digestibility (hydrolysis index, resistant starch, slowly digestible starch) – Structural interactions (starch–phenolic and protein–phenolic interactions) – Nutritional properties (digestibility, glycemic index estimation)	Microwave heat–moisture treatment lowered the in vitro starch hydrolysis index of sorghum meal without added phenolics from 69.89% to 54.33%. Phenolic-containing systems showed lower reported hydrolysis index (35.99–36.18%); findings were derived from in vitro measurements, not postprandial testing.	[55]
Composite flour from wheat and mature unripe pawpaw fruit	Substitution of wheat flour with 10%, 20%, 30%, 40%, and 50% of pawpaw fruit flour in composite formulations	Composite flour for functional food applications	Wheat–pawpaw composite flour; physicochemical and in vitro digestion assays	– Starch digestibility (rapidly digestible, slowly digestible, resistant starch) – Nutritional composition (fiber, ash, moisture, fat) – Functional properties (water absorption, swelling, bulk density) – Antioxidant activity (total phenolics, ABTS, ORAC)	At 50% unripe pawpaw flour substitution, the study reported substantially higher resistant starch and slowly digested starch (2836% and 1321% relative increases versus wheat flour). Water absorption, swelling, compositional measures and in vitro antioxidant indices also differed; this is a composite-flour evaluation.	[56]
Molokhia (*Corchorus olitorius*) leaf powder	Incorporated 0%, 2%, 4%, and 6% of Molokhia into wheat-based flatbread formulation	Molokhia-incorporated flatbread	Formulated flatbread; compositional, in vitro digestion assays and sensory evaluation	– Starch digestibility fractions (rapidly digestible starch, slowly digestible starch, resistant starch) – Nutritional composition (fiber, protein, moisture, fat) – Sensory properties (color, taste, texture, overall acceptability)	Incorporating 2% molokhia leaf powder in flatbread reduced rapidly digestible starch and rapidly available glucose and increased slowly digestible starch, and resistant starch fractions under assay conditions; the starch digestibility index declined from 18.58 to 11.21. Sensory acceptance was highest at 2%, with lower scores at higher inclusion.	[57]
Zucchini polysaccharide	Inclusion 1%, 2%, 3%, 4%, and 5% (*w*/*w*) into potato starch system	Starch–polysaccharide gel system	Potato starch–polysaccharide model; physicochemical and in vitro digestion assays	– Starch digestibility fractions (rapidly digestible starch, slowly digestible starch, resistant starch) – Glycemic index estimation – Rheological properties (viscosity, gel strength) – Structural properties (microstructure, crystallinity, pasting behavior)	Zucchini polysaccharide reduced rapidly digestible starches of potato-starch (25.09% to 16.59%) and increased resistant starch (49.92% to 56.64%); estimated GI declined from 70.89 to 65.22. Viscosity, gel network and freeze–thaw characteristics also changed.	[53]
Kiwifruit soluble and insoluble dietary fiber	Mixed into wheat starch system prior to gelatinization	Starch–dietary fiber composite system	Wheat starch–fiber model; physicochemical and in vitro digestion assays	– Starch digestibility fractions (rapidly digestible starch, slowly digestible starch, resistant starch) – Pasting properties (viscosity, gelatinization behavior) – Structural properties (microstructure, amylose leaching, hydrogen bonding)	Kiwifruit soluble and insoluble fibers reduced in vitro wheat-starch digestibility and amylose leaching in the tested systems. Pasting viscosity and retrogradation depended on fiber type and level.	[6]

Note: Reported findings are specific to the experimental systems indicated. Physicochemical characterization, in vitro digestion and cell-based observations are not evidence of physiological benefit in humans. Estimated glycemic indices are distinct from measured postprandial responses; the human crossover trial is identified separately.

### 3.3. Plant-Derived Dietary Fibers

Plant-derived dietary fibers differ in hydration, adsorption, and structural properties according to their botanical source and processing conditions (Table 3). Isolated rice and cereal—bran fractions showed source-dependent differences in water- and oil-holding capacities, glucose and cholesterol adsorption, and α-amylase inhibition under laboratory conditions [59,60,61]. Wheat bran insoluble fiber also reduced starch digestion in vitro, with enzyme-binding and inhibition assays providing direct evidence for interactions with α-amylase [60]. Processing studies showed that extrusion, ultrasound, enzymatic treatment, and sequential extraction altered soluble fiber yield, composition, and surface properties [7,8,62]. These changes were not uniformly favorable: moderate electron-beam irradiation increased several hydration and adsorption measures, whereas a higher dose reduced selected properties [63]; micronisation increased antioxidant release from peanut skin during simulated digestion but reduced its hydration properties [64]. Physical treatment of clementine pomace also changed pectin-related structural characteristics without establishing their performance in a finished food [65]. In grape—pomace fractions, phenolic release and fermentation-related observations were obtained from in vitro models and indicate potential substrate utilization without confirming a prebiotic effect after consumption [9]. The level of evidence further differs across these materials. Modified wheat—bran fiber was evaluated using physicochemical assays and a HepG2 cell model [8], whereas Ponkan fiber was additionally examined in streptozotocin-induced diabetic mice [66]. Neither experimental system establishes a corresponding response in humans. High-pressure treatment of coconut pomace was associated with altered fiber structure and digestive-enzyme inhibition in vitro [67]. In formulated foods, oat—bran and konjac-containing 3D-printed noodles showed composition-dependent printing and digestion characteristics [68], while grape—pomace- and pecan-shell-enriched bread showed different composition, matrix features, and estimated glycemic indices in simulated digestion [69]. Overall, these studies distinguish the behavior of isolated fiber fractions from the properties measured after food formulation. Assess hydration, adsorption, enzyme interaction, and microbial substrate availability within the specific processing and experimental context; none of these measurements alone establish a physiological benefit after consumption. Responses to dietary fibers incorporated into complete companion-animal diets are considered separately in Section 4. The research related to quantified feeding results following formulation and is unable to independently verify that the physicochemical characteristics noted in individual fiber systems were unaffected during food processing.

**Table 3:** Plant-derived dietary fibers in functional foods and representative evidence supporting processing-related functionality.

Raw Materials	Processing/Incorporation Approach and Level	Food Matrix/Application Form	Experimental System	Measured Endpoints	Reported Findings	Ref.
Corn bran dietary fiber	Twin-screw extrusion, ultrasonic treatment, enzyme hydrolysis; fiber content 4.58–6.54% after modification	Modified soluble dietary fiber ingredient	Physicochemical characterization; in vitro adsorption and antioxidant assays	– Composition (soluble dietary fiber content, monosaccharide composition) – Physicochemical properties (molecular weight, solubility, viscosity) – Structural analysis (Fourier transform infrared spectroscopy, scanning electron microscopy)	Ultrasound and twin-screw extrusion increased soluble dietary fiber from 2.42% to 4.58% and 6.54%, respectively; dual-enzyme treatment further increased recovery. Water/oil retention, nitrite adsorption, and antioxidant assay responses differed with treatment.	[7]
Four rice cultivars (SR-4, K-39, Mushq Budij, Zhag)	Extraction and isolation of dietary fiber fractions from rice cultivars	Isolated dietary fiber ingredient	Isolated-fiber physicochemical characterization; in vitro binding/enzyme assays	– Composition (total, soluble, insoluble dietary fiber) – Physicochemical properties (water-holding, oil-holding capacity) – Functional properties (cholesterol binding, alpha-amylase inhibition) – Structural analysis (Fourier transform infrared spectroscopy, scanning electron microscopy)	Zhag fiber had the highest total dietary fiber (76.90 g/100 g); SR-4 had the highest measured water-holding (1.40 g/g) and oil-holding (5.74 g/g) capacities and cholesterol binding at neutral pH (9.30 mg/g). α-Amylase inhibitory activity differed by cultivar.	[59]
Wheat bran insoluble dietary fiber	Isolation of insoluble dietary fiber and evaluation in starch digestion system	In vitro starch digestion model	In vitro starch digestion and enzyme-binding assays	– Starch digestion (digestion rate, digestible starch fraction) – Enzyme kinetics (alpha-amylase inhibition type) – Molecular interaction (fluorescence quenching, ultraviolet-visible spectroscopy, confocal microscopy)	Wheat-bran insoluble dietary fiber reduced in vitro starch digestion rate and digestible starch content. Kinetic, fluorescence, and imaging analyses supported mixed-type α-amylase inhibition and fiber–enzyme binding in the tested model.	[60]
Insoluble dietary fibers from rice bran, wheat bran, highland barley bran, and tartary buckwheat bran	Extraction and isolation of dietary fiber fractions from cereal brans	Isolated cereal bran dietary fiber ingredients	Physicochemical characterization; in vitro adsorption assays	– Functional properties (glucose adsorption, cholesterol binding, metal binding) – Structural properties (microscopic morphology) – Composition (monosaccharide profile)	Highland barley bran insoluble fiber showed the highest glucose adsorption (3.2 mmol/g), whereas tartary buckwheat bran insoluble fiber had the highest cholesterol adsorption (10.5 mg/g at pH 7.0); fiber morphology and adsorption differed among sources.	[61]
Pinot noir grape pomace dietary fiber–bound polyphenol matrix	Isolation of soluble and insoluble dietary fiber fractions	Fiber-bound polyphenol fractions	Simulated gastrointestinal digestion; in vitro colonic fermentation	– Polyphenol release (gastric and intestinal digestion phases) – Antioxidant activity (radical scavenging assays) – Fermentation characteristics (short-chain fatty acids, microbial response)	Fiber-bound polyphenols were released predominantly during simulated colonic fermentation. Soluble and insoluble fractions differed in phenolic release, antioxidant assays, fermentation metabolites, and microbiota responses; these observations indicate in vitro prebiotic potential only.	[9]
Wheat bran	Bacterial–enzymatic synergistic pretreatment applied to wheat bran	Modified soluble dietary fiber ingredient	Physicochemical and in vitro adsorption assays; HepG2 cell model	– Composition (soluble dietary fiber yield) – Physicochemical properties (water- and oil-holding capacity) – Functional properties (glucose and cholesterol adsorption) – Cellular assays (lipid accumulation, oxidative stress markers) – Structural analysis (Fourier transform infrared spectroscopy, X-ray diffraction, scanning electron microscopy)	Bacterial–enzymatic pretreatment increased wheat-bran soluble fiber yield from 3.12% to 5.72% and altered porosity and adsorption properties. Lower lipid accumulation and oxidative-stress markers were reported in oleic-acid-treated HepG2 cells.	[8]
Distillers’ grains insoluble fiber	Sequential enzyme pretreatment followed by ultrasound–microwave alkaline extraction and bleaching	Processed insoluble dietary fiber ingredient	Physicochemical and structural characterization; in vitro adsorption assays	– Physicochemical properties (water-holding, swelling capacity, cation-exchange capacity) – Functional properties (glucose adsorption capacity) – Structural analysis (scanning electron microscopy, Fourier transform infrared spectroscopy, X-ray diffraction)	Sequential enzyme, ultrasound–microwave, and bleaching steps yielded an insoluble fiber fraction with higher water-holding capacity (4.06 g/g), swelling (3.2 mL/g), and glucose adsorption (25.23 mg/g) in the reported assays.	[62]
Modified Ponkan peel dietary fiber	Alkali treatment combined with homogenization–enzymolysis processing	Modified soluble and insoluble dietary fiber fractions	Physicochemical characterization; in vitro adsorption; diabetic mouse feeding model	– Physicochemical properties (water-holding, oil-holding, glucose adsorption) – Structural properties (porosity, crystallinity) – In vivo outcomes (blood glucose, gut microbiota composition)	Homogenization–enzymolysis increased porosity and selected hydration/adsorption properties of Ponkan fiber. Lower blood glucose and microbiota differences were measured in streptozotocin-induced diabetic mice.	[66]
Electron beam–irradiated wheat bran fiber	Electron-beam irradiation (5–25 kGy); moderate treatment range 5–15 kGy	Modified wheat bran dietary fiber	Physicochemical characterization; in vitro adsorption and radical-scavenging assays	– Composition (soluble and insoluble dietary fiber content) – Physicochemical properties (water-holding, oil-holding capacity) – Structural analysis (Fourier transform infrared spectroscopy, scanning electron microscopy)	Electron-beam treatment at 5–25 kGy affected wheat-bran fiber structure; moderate irradiation (5–15 kGy) increased soluble dietary fiber content (4.83% to 6.40%), water-holding and oil-holding capacities, and measured radical-scavenging activity.	[63]
Micronised peanut skin fiber	Micronisation applied to peanut skin to modify structure and functional properties	Processed insoluble dietary fiber ingredient	Physicochemical and structural characterization; simulated gastrointestinal digestion	– Structural properties (surface morphology, branching structure) – Physicochemical properties (hydration capacity) – Antioxidant release (in vitro digestion model)	Micronisation increased the surface area and release of antioxidant-associated compounds during simulated digestion but reduced the hydration properties of peanut-skin insoluble fiber.	[64]
Clementine pomace dietary fiber and cell wall polysaccharides	Hot air drying, homogenization, freeze drying, and extrusion	Processed fruit pomace fiber ingredient	Compositional, chromatographic, and structural characterization	– Composition (dietary fiber, carotenoids, phenolics) – Structural properties (polysaccharide composition, degree of esterification) – Spectroscopic analysis (chromatography, structural profiling)	Homogenization increased total dietary fiber content, while freeze-dried and extruded pomace showed the highest carotenoid and phenolic contents, respectively. Processing changed soluble cell-wall polysaccharide composition and pectin esterification.	[65]
Coconut pomace dietary fiber	High-pressure homogenization and microfluidization applied to coconut pomace	Processed dietary fiber ingredient	Physicochemical characterization; in vitro enzyme inhibition and binding assays	– Enzyme activity (alpha-amylase inhibition) – Starch digestion (digestibility kinetics) – Structural properties (particle size, morphology)	Dynamic high-pressure microfluidization modified particle size, crystallinity, surface groups and enzyme interactions. The DHPM100 condition produced the strongest reported inhibition of α-amylase and α-glucosidase in vitro; no post-consumption glycemic response was measured.	[67]
Oat bran and konjac flour dietary fiber	Oat bran and konjac flour incorporated at varied proportions; the 6% oat bran/2% konjac formulation was identified as the highest-printability combination	Fiber-enriched 3D-printed chicken noodles	Formulated 3D-printed food; rheology, texture and in vitro digestion	– Rheological properties (viscosity, viscoelasticity) – In vitro digestion (starch and protein digestion kinetics) – Structural properties (printability, texture, water-binding)	Fiber-rich formulations increased dough viscosity and viscoelasticity and prolonged starch/protein digestion in vitro. The 6% oat bran plus 2% konjac formulation showed the highest printability; konjac above 4% adversely affected cooking quality and texture.	[68]
Grape pomace and pecan shell dietary fiber	Incorporated into bread formulations as composite fiber-rich ingredients – Grape pomace: 0%, 5%, 8% – Pecan shell: 0%, 2%, 5%	Fiber-enriched baked bread	Formulated bread; physicochemical assays and simulated in vitro digestion	– Nutritional composition (fiber, phenolics, protein, minerals) – In vitro digestion (glycemic index, sugar release) – Structural properties (scanning electron microscopy, Fourier transform infrared spectroscopy) – Bioaccessibility (phenolic compound release)	Bread formulations containing grape pomace and pecan shell had higher dietary fiber, minerals, protein and phenolic contents than control bread. In vitro digestion yielded lower estimated glycemic index values; microscopy and spectroscopy indicated formulation-dependent matrix differences, without measured human glycemic responses.	[69]

Note: Reported findings refer to the stated experimental systems. Adsorption and enzyme-inhibition assays, simulated digestion or fermentation, cell studies, and rodent experiments represent different levels of evidence. Evaluation of a formulated food does not establish a human physiological benefit unless the relevant outcome was measured in humans.

### 3.4. Plant Prebiotic Fibers and Oligosaccharides

The carbohydrates considered here overlap with the dietary fibers discussed in Section 3.3 but are grouped by studies examining oligosaccharide production, microbial substrate use, and fermentation-related measurements (Table 4). Their experimental scope is heterogeneous. Enzymatic and hydrothermal treatment of sugarcane straw, coffee husk, defatted rice bran, corncob, and pineapple leaves yielded oligosaccharide preparations with different compositions and recoveries [10,70,71,72]. However, production yield and resistance to simulated digestion differ from selective microbial consumption; certain investigations have identified the carbohydrate products without assessing fermentation or host reactions. Garlic fructans and neutral oligosaccharides isolated from apple pectin supported growth of selected bacterial strains in vitro, with outcomes dependent on the fractions and microorganisms tested [11,73]. Oat β-glucan showed changes in molecular size and microbial composition in a porcine colonic fermentation model [13], while pectin from sweet potato leaves was evaluated mainly for hydration, emulsion stability, and starch digestion in formulated noodles [74]. These observations represent different aspects of carbohydrate functionality and cannot be assigned a common level of physiological evidence. Studies of finished foods provide a complementary formulation context. Yacón syrup incorporated into a fruit coating contributed fructooligosaccharides and affected product properties during refrigerated storage [75]. In cereal flour, fermented Riceberry rice and fruit- or sugarcane-based beverages, β-glucan, inulin, and other carbohydrate preparations were assessed for bacterial growth, probiotic viability, processing behavior, or sensory characteristics [76,77,78,79,80]. Responses were not uniform: in fermented sugarcane juice, inulin increased the viability of *Lacticaseibacillus rhamnosus* at selected stages but did not significantly increase that of *Lactiplantibacillus plantarum* [80]. Extracted inulin and fructooligosaccharides also varied by botanical source and extraction conditions [12]. These findings show that carbohydrate identity, processing, and the formulation environment matter for the measured endpoints. Prebiotic potential inferred from microbial culture or fermentation assays remains separate from demonstrated physiological benefits following consumption. Companion-animal feeding evidence is discussed in Section 4 as an application of these ingredients within complete diets. The observed microbial, fermentation, and fecal responses cannot be taken as direct confirmation that an isolated carbohydrate’s structural or in vitro properties persist after formulation.

**Table 4:** Plant prebiotic fibers and oligosaccharides in functional foods and representative evidence supporting processing-related functionality.

Raw Materials	Processing/Incorporation Approach and Level	Food Matrix/Application Form	Experimental System	Measured Endpoints	Reported Findings	Ref.
Sugarcane straw and coffee husk arabinoxylan	Alkaline extraction and enzymatic hydrolysis; extraction yield 40.35%; xylooligosaccharides 10.23 g/L (sugarcane), 8.45 g/L (coffee husk)	Oligosaccharide-rich extract	Process optimization; compositional characterization	– Starch digestibility fractions (rapidly digestible starch, slowly digestible starch, resistant starch) – Pasting properties (viscosity, gelatinization behavior) – Structural properties (microstructure, amylose leaching, hydrogen bonding)	Optimized extraction yielded approximately 40.35% arabinoxylan; enzyme mixtures produced 10.23 g/L xylooligosaccharides from sugarcane straw and 8.45 g/L from coffee husk.	[10]
Corncob-derived xylooligosaccharides	Enzymatic preparation of corncob xylooligosaccharides; single-dose oral toxicity assessment at 5000 mg/kg body weight	Prebiotic oligosaccharide preparation	In vitro bacterial-culture and antioxidant assays; single-dose acute oral toxicity in rats	– Prebiotic activity (probiotic growth stimulation) – Antioxidant activity (radical scavenging, reducing power) – Toxicity assessment (body weight, organ weight, blood parameters)	Corncob xylooligosaccharides supported growth and β-xylosidase activity in the *Lactobacillus* strains tested; antioxidant responses were observed in DPPH, ABTS and FRAP assays. No significant acute toxicity was reported at a single oral dose of 5000 mg/kg body weight in rats; this is not evidence of long-term benefit.	[70]
Defatted rice bran	Alkaline extraction and xylanolysis; arabinoxylan 31.27%; oligosaccharides 13.80%	Oligosaccharide-rich extract	In vitro simulated digestion; bacterial-culture fermentation	– Digestive resistance (in vitro gastrointestinal model) – Fermentation metabolites (short-chain fatty acids, organic acids) – Microbial growth (lactic acid bacteria proliferation)	Under optimized extraction/hydrolysis conditions, arabino-xylooligosaccharides yield was 13.80%; over 80% remained after simulated digestion. Tested lactic acid bacterial cultures produced acetic, propionic and lactic acids under the experimental conditions.	[71]
Pineapple leaves	Hydrothermal treatment 160–190 °C (20–90 min) and enzymatic hydrolysis; yield 23.7% xylooligosaccharides	Oligosaccharide mixture derived from lignocellulosic biomass	Hydrothermal and enzymatic process optimization; product composition	– Oligosaccharide yield (xylooligosaccharides, glucooligosaccharides, cellobiose) – Process parameters (temperature, time, inhibitor concentration) – Biomass conversion efficiency	Hydrothermal pretreatment at 160 °C for 60 min gave reported xylooligosaccharide and glucooligosaccharides yields of 23.7% and 18.3%; subsequent controlled cellulase treatment yielded approximately 174 mg cellobiose/g dry biomass. Prebiotic activity was not evaluated.	[72]
Fructooligosaccharide syrup (yacón)	Yacón syrup incorporated at 10% into gelatin coating; melon storage evaluated for 6 days at 10 °C	Coated fresh-cut fruit	Coated-fruit storage trial; physicochemical, sensory and microbiological evaluation	– Physicochemical properties (moisture, firmness, color stability) – Storage stability (shelf-life changes over time) – Functional composition (fructooligosaccharide content)	Yacón syrup contained 17.22% fructooligosaccharide. Incorporation of 10% syrup modified coating properties; coated minimally processed melon was evaluated over 6 days at 10 °C, with quality preservation reported for 5 days.	[75]
Garlic-derived oligosaccharides	Acid hydrolysis of garlic polysaccharides (degree of polymerization <10) followed by purification and ultrafiltration	Oligosaccharide mixture	In vitro acid-resistance and selected bacterial-growth assays	– Prebiotic activity (probiotic growth stimulation) – Digestive resistance (simulated gastric conditions) – Structural properties (degree of polymerization, molecular weight)	Acid hydrolysis increased the proportion of garlic oligosaccharides with DP <10 from 15% to 75%. The oligosaccharide preparation showed greater growth support for the tested *lactobacilli* than the parent polysaccharide in vitro.	[11]
Apple pectin	Extraction and chromatographic fractionation; structure-dependent fractions	Purified oligosaccharide fractions	Oligosaccharide structural characterization; selected bacterial-growth assays	– Oligosaccharide structure (chromatography, molecular characterization) – Prebiotic activity (growth of probiotic bacteria) – Functional composition (degree of polymerization, linkage type)	Purified neutral oligosaccharides (DP 3–13) from apple pectin supported growth of selected *Lactobacillus* strains in vitro.	[73]
Sweet potato leaf pectin	Extraction; galacturonic acid 55.21%; methylation 61.60%	Hydrocolloid ingredient	Ingredient physicochemical characterization; formulated-noodle in vitro digestion	– Physicochemical properties (composition, methylation, acetylation) – Functional properties (water-holding capacity, emulsion stability) – Starch digestibility (in vitro digestion kinetics)	Extracted sweet potato leaf pectin contained 55.21 ± 2.57% galacturonic acid and 61.60% methylation. Higher water-holding capacity and emulsion stability index than comparator oat β-glucan were reported, with dose-dependent changes in in vitro starch digestion in formulated noodles.	[74]
Oat β-glucan	Direct use as dietary fiber substrate in in vitro fecal fermentation model (0–48 h)	Purified polysaccharide substrate	In vitro porcine colonic fermentation	– Microbiota composition (relative abundance of beneficial and harmful bacteria) – Structural properties (molecular weight, glycosidic linkage changes) – Fermentation characteristics (degradation kinetics)	Oat β-glucan molecular weight decreased during in vitro porcine colonic fermentation; microbial composition, including *Lactobacillus* abundance, differed from comparator fibers under the tested conditions.	[13]
β-glucan and *Bacillus coagulans* in cereal flour matrix	β-Glucan and *Bacillus coagulans* incorporated into cereal flours; storage evaluated for 150 days	Functional cereal flour and porridge product	Cereal flour/porridge formulation; storage, texture and sensory evaluation	– Microbiological stability (probiotic viability during storage) – Texture and rheology (hardness, adhesiveness, cohesiveness, gumminess) – Sensory acceptability (consumer evaluation)	β-Glucan addition did not significantly alter *Bacillus coagulans* viability during 150 days of flour storage (above 7.45 log CFU/g in the synbiotic flour). Flour and porridge textural characteristics differed among formulations; sensory acceptance was highest for the probiotic-only formulation.	[76]
Riceberry rice	Germination (3–4 days) followed by fungal fermentation (*Pleurotus ostreatus*)	Fermented rice substrate	Germination and fungal fermentation; selected bacterial-growth assays	– Prebiotic activity (growth of probiotic and pathogenic bacteria) – Bioactive compounds (gamma-aminobutyric acid, β-glucan content) – Fermentation kinetics (time-dependent changes)	Germination and subsequent *Pleurotus ostreatus* fermentation changed GABA and β-glucan content and supported growth of selected bacterial test strains. Growth-based prebiotic scores were obtained in vitro; no consumer intervention was conducted.	[77]
Riceberry rice	Solid-state fermentation of germinated rice using mushroom mycelium (*Pleurotus ostreatus*)	Crude β-glucan extract (cell-based cytotoxicity assays and in vitro prebiotic assays)	Solid-state fermentation and extraction; bacterial growth and cell-line assays	– Prebiotic activity (probiotic growth, prebiotic index values) – Cytotoxicity (cancer cell viability, apoptosis induction, normal cell safety) – Structural characterization (Fourier-transform infrared spectroscopy, thermogravimetric analysis)	Fermentation-derived β-glucan extract supported growth of *Lacticaseibacillus rhamnosus* and *Bacillus coagulans* in culture; reduced SW480 colorectal cancer-cell viability was reported in a separate cell-line assay.	[78]
Chicory root, Jerusalem artichoke, globe artichoke	Aqueous extraction optimized by response surface methodology; temperature 60–80 °C, time 20–60 min, solvent-to-solid ratio 10–40 mL/g	Extracted inulin and fructooligosaccharide ingredient	Response-surface extraction optimization; HPLC/FTIR characterization	– Extraction yield and efficiency – Chemical composition (purity, degree of polymerization) – Process parameters (temperature, extraction method)	Extraction optima and inulin plus fructo-oligosaccharides recovery varied by plant material. Reported optimized contents were 70.5 g/100 g dry weight for chicory root and 81.1 g/100 g for Jerusalem artichoke.	[12]
Inulin and beta-glucan	Fortification of Alphonso mango juice; Inulin: 31.89 mg/mL; β-glucan: 19.93 mg/mL; Storage: up to 8 weeks at 4 °C	Functional beverage	Fermented juice formulation and refrigerated storage; probiotic viability and sensory evaluation	– Probiotic viability (log colony-forming units per milliliter during storage) – Sensory evaluation (acceptability, flavor stability) – Chemical profile (volatile compounds by gas chromatography–mass spectrometry)	Optimized mango-juice fortification used 31.89 mg/mL inulin and 19.93 mg/mL β-glucan. *Pediococcus pentosaceus* viability was higher than in the unfortified comparator during selected refrigerated storage points; sensory acceptability was retained through week 6.	[79]
Sugarcane juice with inulin and fructooligosaccharides	Fermentation with *Lactiplantibacillus plantarum* (7.5 h) and *Lacticaseibacillus rhamnosus* (8.5 h); inulin supplementation 1–2% (*w*/*v*); storage at 4 °C for 2 weeks	Fermented sugarcane beverage	Fermented juice formulation and refrigerated storage; strain-specific viability evaluation	– Probiotic viability (CFU during fermentation and storage) – Fermentation characteristics (pH, acidity changes) – Product stability (storage performance)	Inulin supplementation increased *Lacticaseibacillus rhamnosus* viability at specific post-fermentation/storage time points; corresponding significant gains were not found for *Lactiplantibacillus plantarum*. Both strains remained near 8 log CFU/mL under the reported processing/storage conditions.	[80]

Note: Reported findings are specific to the experimental systems used, including ingredient characterization, simulated digestion, microbial culture or fermentation, cell-based assays, formulated-food evaluation, and animal toxicity testing. Microbial growth, probiotic viability in food products, and in vitro fermentation do not, by themselves, establish prebiotic effects following consumption. Acute toxicity assessments provide safety-related information but do not establish physiological efficacy.

### 3.5. Fermented Plant-Derived Ingredients

Fermentation modifies plant substrates through microbial metabolism, although the direction of compositional and technological changes depends on the substrate and organism. In the studies summarized in Table 5, solid-state fungal fermentation altered protein and amino acid composition, phenolic profiles, antinutritional constituents, and microstructure in legumes and cereal matrices [2,4,14,81,82,83]. The effects were not uniformly favorable: *Rhizopus*-fermented legumes showed reductions in phytate alongside increases in trypsin inhibition [2], while changes in phenolic and antioxidant measurements varied by substrate and assay [2,83]. Fermentation of cashew by-product also changed protein digestibility and technological properties, but the resulting powder did not exhibit gel formation under the conditions evaluated [84]. These observations distinguish changes in ingredient composition from technological performance in a finished food. Lactic acid fermentation provided a different set of processing responses in legume and beverage systems. Changes in viable cell counts, peptide fractions, phenolic measurements, acidity and physical properties depended on bacterial strain, fermentation conditions, and the plant substrate [85,86,87,88]. In navy bean fermentation, mixing conditions affected microbial growth, whereas water absorption, oil binding, and foam capacity decreased after processing [85]. The production of riboflavin, dextran, and oligosaccharides in fermented plant beverages provides evidence of metabolite formation within a formulated system, but not of a prebiotic response after consumption [89]. Fermented pea yoghurt and tomato were also evaluated in mouse disease models, with reported inflammatory, metabolic, and microbiota responses that should be distinguished from physicochemical characterization and from human intervention evidence [88,90]. Cell-based observations for fermented chicory by-products [91] and a mouse toxic-injury model for fermented plant beverages [92] add model-specific findings without establishing clinical effects. The research shows that fermentation can alter both nutritional content and technological characteristics, but the effectiveness must be assessed within the specific food matrix and experimental setup being investigated. The use of fermented plant ingredients in comprehensive diets for companion animals is discussed in Section 4, where the effects of feeding are analyzed independently from the technological and compositional modifications observed in studies involving human food.

**Table 5:** Fermented plant-derived ingredients in functional foods and representative evidence supporting processing-related functionality.

Raw Materials	Processing/Incorporation Approach and Level	Food Matrix/Application Form	Experimental System	Measured Endpoints	Reported Findings	Ref.
**Ingredient pre-fermentation**						
Chickpea, pigeon pea, soybean	*Rhizopus oligosporus* solid-state fermentation; 34 °C, 52 h; dried 45 °C 16–18 h	Fermented legume flour	Compositional and physicochemical characterization; in vitro antioxidant assays	– Antioxidant activity (radical scavenging, ferric reducing antioxidant power, metal chelation) – Antinutrients (phytic acid, tannins, saponins, trypsin inhibitors) – Mineral composition and bioavailability – Structural characterization (Fourier-transform infrared spectroscopy)	Phenolic content and antioxidant assay responses increased for the two chickpea types and soybean, but responses in pigeon pea were non-significant. Phytic acid decreased in all fermented legumes, whereas trypsin inhibition increased; changes in tannins and saponins depended on legume type.	[2]
Chickpea, pigeon pea, soybean	*Rhizopus oligosporus* solid-state fermentation	Fermented legume matrix	Compositional, technological and microstructural characterization	– Nutritional composition (protein, amino acids, ash content) – Functional properties (water absorption, emulsification, colour parameters) – Microstructure (scanning electron microscopy)	Fermentation was associated with higher measured protein (5–14%) and ash (5–15%) contents and changes in amino acid composition, color, technological properties and microstructure across the legumes tested.	[4]
Cashew by-product	*Rhizopus oligosporus* solid-state fermentation; 28 °C, 10^8^ spores/mL, 32 h; dried 70 °C	Fermented plant-based powder	Physicochemical characterization and in vitro protein digestion	– Nutritional composition (protein, lipid content) – Antioxidant activity (DPPH, ferric reducing antioxidant power assays) – Digestibility (in vitro protein digestibility) – Functional properties (technological characteristics of dried ingredient)	At the selected fermentation condition (28 °C, 108 spores/mL, 32 h), protein content, in vitro protein digestibility and DPPH/FRAP assay responses increased. The dried powder did not form a gel under the tested conditions.	[84]
Lentils, chickpeas, soybeans	*Pleurotus ostreatus* solid-state fermentation	Fermented legume substrate	Compositional analysis, in vitro protein digestion and antioxidant assay	– Nutritional composition (protein content, digestibility) – Antinutrients (phytate, tannins) – Antioxidant activity (DPPH radical scavenging)	*P. ostreatus* fermentation of lentil, chickpea and soybean substrates was associated with higher measured protein content and in vitro digestibility, lower phytate and tannin contents and increased DPPH radical-scavenging activity.	[81]
Green kernel black beans	*Eurotium cristatum* solid-state fermentation; time-course monitoring (dynamic fermentation; specific temperature)	Fermented legume product	Time-course compositional, sensory and metabolomic analyses	– Phenolic composition (including flavonoids and isoflavones) – Amino acids (17 amino acids including branched-chain amino acids) – Metabolomics (51 differential metabolites, pathway analysis) – Sensory properties (aroma profile, sourness reduction)	Fermentation increased measured polyphenols and flavonoids, changed isoflavone forms and amino acid profiles, and reduced soluble protein, polysaccharide and short-chain fatty acid contents in the fermented beans.	[14]
Wheat, rice, wheat flour	*Ganoderma lucidum* solid-state fermentation	Fermented grain-based ingredient	Fermented grains; compositional, safety-related and in vitro bioactivity assays	– Nutritional composition (protein, vitamins, polysaccharides) – Phytochemical composition (triterpenes) – Functional properties (antioxidant, anti-inflammatory activity) – Safety assessment (heavy metal content)	Fermented grain composition varied by substrate; protein, polysaccharide and selected vitamin contents increased. Heavy-metal concentrations were lower in tested samples, and antioxidant/anti-inflammatory assay responses were reported; these measurements do not independently establish consumer safety or clinical effects.	[82]
Corn silk	*Aspergillus niger* solid-state fermentation; 10 days	Fermented plant residue	Fermented corn silk; chromatographic and in vitro antioxidant assays	– Phenolic profile (individual phenolic compounds by chromatography) – Antioxidant activity (ABTS, DPPH, ferric reducing antioxidant power) – Metabolite changes (organic acids, sugars)	During fermentation, total phenolic and flavonoid measurements increased, and antioxidant responses increased in ABTS, DPPH, and FRAP assays but not substantially in CUPRAC. Chromatographic profiles and organic acids also changed.	[83]
**Fermentation during food manufacture**						
Navy bean slurry	*Lactobacillus paracasei* CBA L74 fermentation with optimized mixing and aeration conditions during fermentation	Fermented legume slurry	Fermented navy bean suspension; fermentation kinetics and physicochemical assays	– Microbial growth (colony-forming units, biomass increase) – Fermentation characteristics (pH, acidification kinetics) – Physicochemical properties (viscosity, composition changes)	Optimized mixing supported faster bacterial growth; fermentation reduced water absorption, oil binding, and foaming capacities of resulting powders. Phenolic and antioxidant changes were attributed mainly to prior sterilization.	[85]
Chickpea flour	Multiple LAB strains fermentation of 20% (*w*/*w*) chickpea puree for 48 h using; final pH ≤5 followed by freeze-drying	Fermented chickpea flour	Fermented chickpea flours; compositional, enzyme-inhibitory and in vitro antioxidant assays	– Bioactive compounds (bioactive peptides, free amino groups, polyphenols) – Antinutrients (phytic acid) – Antioxidant activity (total antioxidant capacity) – Fermentation characteristics (pH reduction)	Responses differed among strains: selected cultures increased free amino groups and enzyme-inhibitory peptide fractions; others showed greater phenolics and in vitro antioxidant activity with lower phytate.	[86]
Chickpea, green, and red lentil beverages	*Lactiplantibacillus plantarum* 299v fermentation; 24–48 h	Fermented legume beverage	Fermented legume beverages; physicochemical characterization, antioxidant assays and protein profiling	– Antioxidant activity (radical scavenging capacity) – Protein profile (peptide composition, proteolysis) – Fermentation characteristics (pH reduction, metabolic activity)	Fermentation changed pH, fiber/protein measurements and antioxidant assays in chickpea and lentil beverages. Antioxidant assay responses differed by substrate and fermentation duration.	[87]
**Fermented food evaluation (animal model or product characterization)**						
Pea-based yoghurt	*Lactiplantibacillus plantarum* fermentation; dietary administration in dextran sulfate sodium-induced colitis mouse model	Fermented pea-based yoghurt	Fermented pea yoghurt; DSS-induced colitis mouse feeding experiment	– Inflammation markers (cytokines, tissue damage, immune organ index) – Microbiota composition (relative abundance of taxa) – Intestinal metabolites (short-chain fatty acids)	In DSS-induced colitis mice, fermented pea yoghurt feeding was associated with lower measured tissue injury and inflammatory indicators, differences in microbial relative abundances and higher reported short-chain fatty acid concentrations; these are mouse-model findings.	[88]
Oat, soy, rice-based substrates	Fermentation using *Weissella confusa* with in situ production of riboflavin and dextran	Fermented plant-based beverage	Fermented plant-based beverages; fermentation and refrigerated-storage evaluation	– Metabolite production (riboflavin, dextran, oligosaccharides, mannitol) – Fermentation characteristics (pH reduction, acidification) – Storage stability (cell viability over time)	*W. confusa* FS54 B2 produced riboflavin, dextran and selected oligosaccharides in plant beverages, with the oat-based substrate giving the strongest reported production profile. Fermentation acidity and viable counts were assessed during storage; no host prebiotic effect was tested.	[89]
Tomato	Probiotic fermentation; dietary intervention in high-fat diet-induced obesity mouse model	Fermented tomato ingredient	Fermented tomato; high-fat-diet mouse feeding experiment, microbiota and metabolomics	– Metabolic markers (body weight, lipid profile, glucose regulation) – Microbiota composition (relative abundance, diversity indices) – Serum metabolomics (lipids, amino acids, metabolic pathways)	In high-fat-diet-fed mice, fermented tomato preparations were associated with differences in body-weight gain, adiposity, serum markers, microbiota profiles and metabolomic measurements relative to comparison groups; these results are specific to the mouse model.	[90]
**Post-fermentation ingredient/product evaluation**						
Chicory by-products	*Lactiplantibacillus plantarum* fermentation with selected strains under controlled fermentation conditions	Fermented chicory-derived ingredient	Fermented chicory by-products; biochemical antioxidant and cultured-cell assays	– Antioxidant activity (DPPH assay, cellular reactive oxygen species assays) – Bioactive compounds (polyphenols, metabolite profiling) – Cell-based assays (hepatic and intestinal cell lines)	Fermentation responses depended on chicory by-product and bacterial strain. Selected fermented endive-peeling preparations reduced measured ROS in HepG2/Caco-2 cell assays, while phenolic/metabolite profiles differed.	[91]
Aromatic and medicinal plant extracts	Plant-beverage fermentation; administration in CCl_4_-treated mice	Fermented plant beverage	Fermented plant beverages; CC_l4_-induced liver/kidney injury mouse model and compositional assays	– Biochemical markers (liver and kidney function indicators) – Antioxidant activity (enzyme activity, radical scavenging) – Histopathology (liver and kidney tissue structure) – Phenolic composition (including flavonoids and tannins)	Fermented plant beverages differed in phenolic and volatile composition and antioxidant assays. In CC_l4_-exposed mice, treated groups showed differences in liver/kidney biochemical indices, antioxidant enzyme activities and histopathological observations.	[92]

Note: Experimental systems identify the setting in which each finding was obtained. Ingredient composition, physicochemical properties, antioxidant assays, in vitro digestion, cellular responses and animal-model findings are not equivalent to demonstrated physiological benefits in human consumers. Fermentation-stage groupings indicate the point of processing or evaluation, not the strength of evidence.

## 4. Application of Plant-Derived Functionality in Companion-Animal Foods

**Figure 2:**
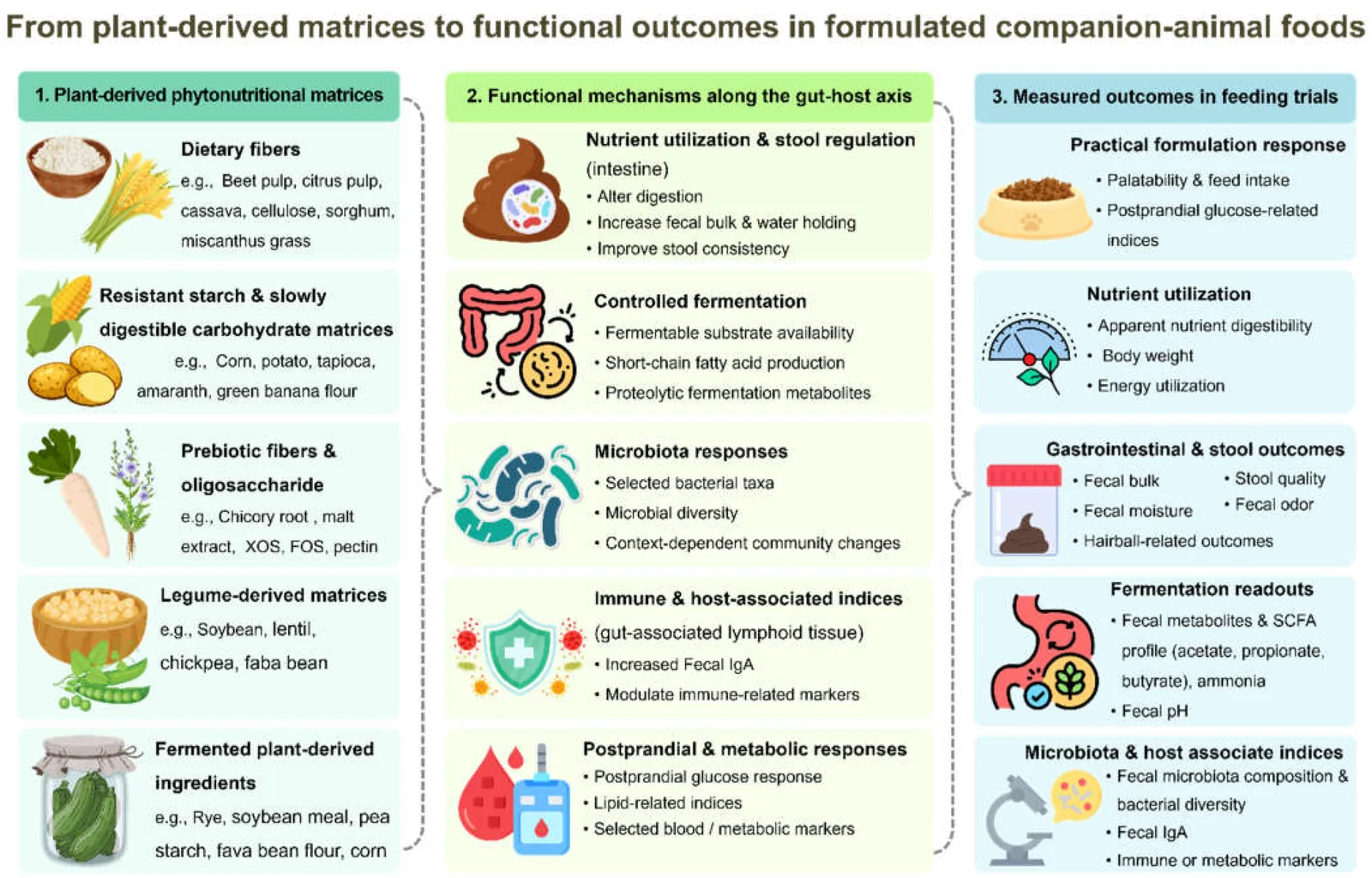
Conceptual framework relating processing-responsive plant-derived matrices to formulation characteristics and measured outcomes in companion-animal feeding studies. Abbreviations: XOS, xylooligosaccharide; FOS, fructooligosaccharide; IgA, immunoglobulin A; SCFA, short-chain fatty acids.

The processing-related properties discussed in Section 3 provide a basis for examining plant-derived ingredients after their incorporation into complete companion-animal diets. Feeding studies evaluate responses to formulated foods under specified dietary conditions; they do not necessarily confirm that individual structural features observed before manufacture remain unchanged following extrusion, cooking, or fermentation. This section considers nutrient utilization, fecal characteristics, colonic fermentation, microbiota responses, postprandial glycemic responses, and diet acceptance in dogs and cats (Table 6 and Table 7). Differences among studies are interpreted with reference to ingredient form, inclusion level, comparator, processing history, and the complete dietary formulation. The evidence is application-specific and is not used to predict physiological responses to human functional foods. Figure 2 summarizes the relationships among plant-derived matrix properties, processing and formulation, and the outcomes evaluated in companion-animal feeding studies.

### 4.1. Nutrient Utilization and Stool Characteristics

Apparent nutrient digestibility and fecal characteristics have been examined across a broad range of plant-derived matrices, but neither endpoint can be inferred reliably from total fiber or starch content alone. In dog diets, cellulose, lignocellulose, Miscanthus grass, guava fiber, and sorghum or rye fractions were associated with different patterns of fecal output, consistency, transit, and apparent nutrient digestibility [29,93,94,95,96,97,98]. These comparisons involved different ingredient substitutions and dietary formulations. Structural fiber inclusion was sometimes associated with greater fecal output or lower apparent digestibility, although similar responses were not observed across all sources or inclusion levels. Trials involving cassava and citrus fibers or blended fiber ingredients reported distinct digestibility and fecal responses, with the accompanying fermentation findings depending on the source and comparator [99,100,101,102,103,104,105]. Legume-derived protein–carbohydrate matrices also require diet-level interpretation. While other legume substitutes produced similar nutrient consumption or stool characteristics, the introduction of soybeans and faba beans decreased specific apparent digestibility coefficients in some formulations [98,106,107,108,109,110,111,112]. Fermented soybean meal was not uniformly more digestible than conventional soybean meal, and the outcomes with fermented plant proteins varied depending on the protein source being replaced and the inclusion level. A different variable is incorporated by processing before diet manufacture [27,113,114,115,116]. In cats, the 15% corn-fermented protein formulation had lower apparent dry matter, organic matter, crude-protein, and gross-energy digestibility than diets containing 0–10%, despite similar stool scores [117]. These findings support evaluation of digestibility alongside fecal output and food composition, without attributing the observed differences to a single ingredient component.

### 4.2. Colonic Fermentation and Fecal Metabolite Profiles

Fecal short-chain fatty acids, branched-chain fatty acids, ammonia, and pH provide information about fermentation-related responses to formulated diets. Their concentrations, however, reflect both metabolite production and subsequent utilization or absorption, and should not be equated with rates of colonic production. Trials involving resistant starch, chicory-derived carbohydrates, citrus or cassava fiber, and prebiotic-containing blends reported differences in selected fecal metabolites, but their directions depended on dietary context and the specific comparator [99,100,101,102,103,104,118,119,120,121,122,123,124]. For example, the chicory studies did not show the same pattern of fecal short-chain fatty acid responses across basal diets, while some fiber blends altered acetate or propionate without corresponding differences in microbial diversity [121,122,123,124]. Fermentation-related responses were also not uniformly greater after ingredient fermentation. Dog studies comparing conventional and fermented soybean products, corn-derived co-products, or graded fermented plant protein found different patterns of fecal metabolites and nutrient utilization [27,114,115,116,125,126,127]. In feline studies, extrusion conditions associated with greater resistant starch preservation were accompanied by higher fecal butyrate and immunoglobulin A in one trial [119], whereas comparisons between resistant-starch-rich and multi-ingredient fiber–prebiotic–probiotic diets produced different microbial and metabolite profiles [120]. Because these are diet comparisons, an observed fecal metabolite difference cannot be assigned exclusively to resistant starch, microbial fermentation of a particular fraction, or fermentation during ingredient production. Fecal profiles are most informative when interpreted with the measured food composition, apparent digestibility, and stool characteristics.

### 4.3. Microbiota and Host-Associated Measurements

Studies using plant-derived fibers, oligosaccharides, resistant starch, and legume-based diets have reported changes in selected fecal taxa, richness, diversity, and community composition [119,120,121,122,123,128,129,130,131]. The directions are not uniform across ingredients or experimental conditions. In cats, xylooligosaccharide supplementation at 0.04% and 0.40% yielded different microbial profiles, and changes in individual genera did not by themselves demonstrate a host-health benefit [131]. Similarly, resistant-starch and multi-ingredient fiber formulations changed different microbial and metabolite endpoints [119,120]. The terms microbiota responses and taxonomic differences are therefore more appropriate here than claims of generalized microbiota improvement. A smaller number of studies examined host-associated endpoints, including fecal immunoglobulin A, circulating immune-cell measurements, inflammatory or oxidative markers, blood lipids, taurine-related variables, and selected cardiac indices [27,110,111,112,119,124,126,132]. It is not appropriate to combine these results as a standard indicator of physiological improvement. Taurine-related and cardiac measurements require separate consideration because of concerns regarding diet-associated dilated cardiomyopathy (DCM) in dogs. Quilliam et al. (2021) [108] evaluated taurine status, nutrient digestibility, and glycemic responses following 7 days of feeding diets containing different legume flours. In a subsequent 28-day trial, Quilliam et al. (2023) [106] assessed taurine status, nutrient and amino acid digestibility, and selected cardiac indices in dogs receiving rice-, lentil-, or wrinkled pea-based diets. Reis et al. (2021) [127] also examined cardiovascular measurements and fecal bile acid excretion in dogs fed fermented fava bean-based diets over 28 days. These studies differed in processing, formulation, and outcome selection and do not establish a causal relationship between legume inclusion and DCM. Sulfur-containing amino acid availability, bile acid–taurine conjugation, fiber-associated bile acid losses, and antinutritional constituents are relevant considerations, but the contributions of these pathways were not established across the feeding trials. The short intervention periods and overlapping research groups further limit conclusions concerning long-term cardiac outcomes. Multi-ingredient botanical supplements present an additional attribution problem because effects cannot be assigned to a single prebiotic or phytonutritional constituent [132].

### 4.4. Starch Digestibility and Postprandial Glycemic Response

Starch-related responses in complete diets depend on the processed carbohydrate source and the surrounding formulation. Studies in dogs have compared high-amylose starch, legume-derived carbohydrates, resistant-starch-enriched formulations, and commercial diets, reporting differences in starch or nutrient digestibility and, in some comparisons, postprandial glucose or insulin measurements [106,108,118,133,134,135,136,137,138,139,140]. Lower apparent starch digestibility does not necessarily correspond to a lower postprandial glycemic response, and neither outcome alone establishes a sustained metabolic benefit. Comparisons of processing methods further indicate that greater resistant starch preservation did not invariably produce stronger fecal fermentation responses [118,133]. Feline data require equally careful interpretation. In a 28-day comparison of complete diets based on potato, sweet potato, cassava, rice, or wheat, glycemic measures were numerically lower for the cassava formulation, but the reported differences were not statistically significant [141]. In studies using *Candida utilis*-fermented pea starch, fermentation influenced product acceptance and selected digestibility or metabolic endpoints, yet postprandial glycemic responses to the fermented and unfermented formulations were not clearly different under the tested conditions [142]. These results do not establish a common glycemic effect of legume starch fermentation across species. Apparent digestibility, postprandial responses, and longer-term metabolic status remain distinct levels of evidence.

### 4.5. Palatability and Formulation Constraints

The application of plant-derived matrices depends on technological performance and acceptance of the complete food, as well as measured responses after feeding. Ingredient replacement can alter kibble expansion, texture, moisture, stool characteristics, and voluntary intake; these variables are rarely isolated from the broader formulation. Fiber-containing diets based on cassava, guava, or other plant co-products were acceptable in the tested formulations, although responses varied with inclusion level and comparator [93,99,100,101,105]. Similarly, legume-based diets showed differences in acceptance and apparent nutrient utilization according to replacement strategy [98,106,107,108,109,110,111,112]. Fermented products make these trade-offs particularly visible. In cat diets containing corn-fermented protein, the 5% formulation was preferred over the soybean-meal control, whereas a corresponding preference was not reported at 10% or 15%; at 15%, selected apparent nutrient digestibility coefficients were lower [117]. In a separate feline study, graded fermented plant protein affected kibble expansion, crude-protein digestibility, fecal ammonia, and intake preference differently across inclusion levels [113]. Studies of fermented pea starch in dogs and cats also separated palatability findings from postprandial and subsequent metabolic or fecal measurements [27,142]. Thus, improved acceptance in one comparison does not demonstrate superior nutritional or physiological performance, and a processing treatment that changes ingredient composition should not be assumed to confer the same response after formulation.

**Table 6:** Translation of plant-derived functional properties to measured responses in dog feeding trials.

Matrix Category	Formulation/Processing Context	Measured Outcome Domains	Synthesis of Reported Responses	Ref.
Legume-derived protein–carbohydrate matrices	Extruded diets with graded soybean or faba-bean inclusion; lentil substitution; pulse-based formulations; raw-meat mixed diets	Apparent nutrient digestibility, fecal characteristics, postprandial glucose, palatability, selected cardiac/taurine indices and microbiota	– Responses differed with ingredient and diet design. – Soybean/faba-bean inclusion reduced selected apparent digestibility coefficients, whereas other plant-protein replacements retained comparable digestibility. – Short-duration pulse trials reported differences in postprandial glucose and amino-acid availability, but do not establish long-term metabolic or cardiac effects.	[98,106,107,108,109,110,111,112]
Resistant and slowly digestible carbohydrates	Extrusion shear, pelleting/baking, high-amylose rice or corn resistant starch, different starch sources, commercial foods, and resistant starch top-dressing	Starch/nutrient digestibility, glycemia, fecal metabolites, microbiota, body-weight change, and estimated gastric emptying	– Processing influenced starch resistance, but higher resistant starch did not reliably correspond to stronger fecal fermentation responses. – Specific formulations differed in postprandial glycemia and body-weight trajectories; some trials reported no glycemic differences. – Resistant starch effects cannot be separated from energy density or whole-diet composition in all comparisons.	[118,128,133,134,135,136,137,138,139,140]
Plant-derived dietary fibers	Extruded or baked complete dog foods; wet-food fiber additions; supplements added to existing diets; some clinical populations. Fiber form, processing, and comparator vary.	Apparent nutrient digestibility, fecal quality/output, fermentation metabolites, fecal microbiota, palatability, transit, and selected clinical endpoints.	– Coarse/low-fermentability fibers and rye often differed in digestibility or stool measures, whereas cassava, citrus, apple pomace, and some blends showed substrate-specific fecal fermentation responses. – Results were not uniform: wheat dextrin produced softer stools without clear SCFA effects; banana resistant starch lowered microbial richness. – Physiological benefits were not established merely from microbial or metabolite shifts.	[26,28,93,94,95,97,99,100,101,102,103,104,105,129,130,143]
Prebiotic fibers and oligosaccharides	Chicory root powder in homemade/cereal-based diets; blends in extruded foods; malt extract and kestose supplements	Nutrient digestibility, fecal SCFAs, microbial counts, stool characteristics, selected immune/metabolic endpoints	– Chicory responses varied with basal cereal and dose: fecal SCFAs increased in one study, but not another. – Fiber blends altered selected fecal metabolites without changing diversity, whereas malt and kestose yielded different microbial/metabolite patterns. – Clinical prebiotic benefits remain incompletely established.	[121,122,123,124,132,144]
Fermented plant-derived ingredients	Fermented rye, soybean meal, natto, pea starch, fava bean, plant protein and corn-derived co-products in complete diets or supplements	Digestibility, extrusion properties, fecal metabolites, postprandial measures, immune-cell assays, palatability and selected blood indices	– Fermentation-associated responses depended on source, formulation and comparator. – Fermented soybean meal was not consistently superior to regular soybean meal; some graded fermented proteins altered crude protein digestibility without fecal SCFA effects. – Pea starch findings include dogs and cats, but feline microbiota were not evaluable. – Short-term immune/cardiovascular measures do not establish clinical benefit.	[27,114,115,116,125,126,127,142]

Note: Measured outcome domains comprise endpoints assessed in the cited dog feeding studies. The synthesis describes diet-level responses under the reported processing, inclusion and comparator conditions; it does not establish that an ingredient property persists unchanged after formulation or that an observed microbiota or metabolite difference confers a health benefit. Individual study designs, doses, comparators and directions of effect are provided in Supplementary Table S1.

**Table 7:** Application of Plant-Derived Functional Properties to Measured Responses in Cat Feeding Trials.

Matrix Category	Formulation/Processing Context	Measured Outcome Domains	Synthesis of Reported Responses	Ref.
Resistant and slowly digestible carbohydrates	High- versus low-shear extruded grain diets with different resistant starch content; resistant-starch-enriched diets compared with fiber–prebiotic–probiotic blends; five complete diets based on potato, sweet potato, cassava, rice or wheat.	Fecal butyrate and other metabolites, microbiota, fecal IgA and stool scores; nutrient digestibility, postprandial glucose and insulin, selected blood lipid measures.	– Low-shear/high-resistant-starch extrusion was associated with higher fecal butyrate and IgA, without a difference in stool firmness. – Resistant starch and multi-ingredient fiber blends produced different fecal microbial and metabolite profiles. – In five-source diet comparisons, cassava-related glycemic differences were numerical and not statistically significant; selected microbiota taxa differed.	[119,120,141]
Plant-derived dietary fibers	Extruded foods containing 1% or 3% orange fiber compared with beet pulp, inulin and no added fiber; Miscanthus grass compared with cellulose or beet pulp; additional Miscanthus–tomato pomace combination.	Apparent nutrient and fiber digestibility, fecal SCFAs, tyramine, stool quality and output, hairball measures, fecal microbiota.	– Orange fiber up to 3% did not change macronutrient digestibility; fecal tyramine was higher with 3% orange fiber than with control or inulin, whereas SCFA contrasts depended on the comparator. – Beet pulp and Miscanthus differed in digestibility and stool scores; most hairball endpoints did not differ. – Beet pulp produced higher fecal acetate/propionate, whereas Miscanthus-containing diets showed greater microbial α-diversity than beet pulp.	[96,145,146]
Prebiotic fibers and oligosaccharides	Complete diets containing 0%, 0.04% or 0.40% xylooligosaccharides (XOS), with cellulose adjusted to maintain the supplemented fraction.	Fecal microbial community, microbial richness, fecal pH, selected circulating acylcarnitines.	– XOS responses were dose dependent. Both doses were associated with differences in selected bacterial taxa; the higher dose showed greater richness and a more distinct microbiota profile. – Neither bacterial changes nor correlations with blood metabolites, alone, demonstrate a host-health benefit.	[131]
Fermented plant-derived ingredients	Extruded cat diets replacing 15% soybean meal with corn-fermented protein at 5%, 10%, or 15%; extruded diets with 5%, 10%, or 15% fermented plant protein replacing soybean meal; and approximately 30% *Candida utilis*-fermented or unfermented pea starch compared with corn-based diets.	Apparent nutrient digestibility, fecal dry matter and output, fecal ammonia and metabolites, kibble expansion, palatability, starch digestibility, postprandial glycemic responses, and selected blood metabolic markers.	– For corn-fermented protein, 15% inclusion reduced apparent dry matter, organic matter, crude-protein, and gross-energy digestibility compared with 0–10%, while 5% was preferred to the soybean-meal control; stool scores did not differ. – At 15% fermented protein, apparent crude-protein digestibility and fecal ammonia were higher, while cats preferred the 10% treatment to the soybean-meal control. – *Candida utilis*-fermented pea starch was preferred to unfermented pea starch without a significant glycemic-response difference. A later feeding study reported greater feline starch digestibility for fermented pea starch, but fecal microbiota could not be quantitatively assessed in cats.	[27,113,117,142]

Note: Reported responses are restricted to measured endpoints in the cited cat feeding studies. The diets differ in processing, ingredient inclusion, comparator, and study design. Changes in fecal metabolites, microbial composition, nutrient digestibility, or selected blood markers should not be interpreted as demonstrated clinical benefits. Studies including both dogs and cats are recorded as separate species-level observations but counted once as publications in Supplementary Table S1. Cases in which feline microbiota could not be evaluated are indicated.

### 4.6. Species-Related Evidence and Limitations

The available dog and cat studies differ in number, ingredient coverage, comparators, processing conditions, feeding duration, and outcome selection (Table 6 and Table 7). This imbalance limits comparison of response magnitude and does not support the claim that either species is generally more or less responsive to plant-derived matrices. In the compiled feline evidence, no eligible legume-derived protein–carbohydrate trial is separately represented in Table 7 as a standalone category; this should be treated as a gap in the present study set, not as proof that such studies do not exist. Differences in the frequency of measured endpoints likewise describe research coverage, not the strength or direction of observed effects. Species physiology provides relevant context but does not substitute for matched dietary comparisons. Differences in carbohydrate digestion and metabolism provide physiological context for interpreting canine and feline feeding studies. Neither species has appreciable salivary amylase activity, while pancreatic starch digestion and glucose metabolism differ between dogs and cats. Genomic and functional evidence indicates adaptation to starch-rich diets during dog domestication [147]. Variation in pancreatic amylase gene (AMY2B) copy number has also been associated with serum amylase activity and differs among dog breeds and individuals [148]. Cats, as obligate carnivores, have limited hepatic glucokinase activity [feline physiology reference required]. High apparent starch digestibility in feline diets is therefore not necessarily inconsistent with differences in postprandial glucose handling. These physiological distinctions provide context for interpreting feeding responses but do not establish species-specific differences in responses to individual plant ingredients. Two studies of *Candida utilis*-fermented pea starch included both dogs and cats and permit comparisons under related formulations [27,142]. In the subsequent feeding study, feline fecal microbiota could not be quantitatively assessed because of sample-quality limitations, preventing a matched microbiota comparison [27]. These publications also used related research animals and formulations, so they should not automatically be regarded as independent replication. Interpretation across species should remain restricted to endpoints measured under sufficiently similar conditions. The companion-animal evidence therefore describes application-specific responses to finished diets, while the human-food processing–matrix framework remains the principal focus of this review.

## 5. Conclusions

Processing-related changes in plant-derived food matrices provide a basis for understanding their nutritional and technological properties in functional-food development. Across the five matrix categories examined, thermal, mechanical, enzymatic, extraction, and fermentation processes affected protein hydrolysis, starch organization, fiber structure, microbial substrate availability, and physicochemical properties. These responses differed among ingredients and processing conditions, and findings from isolated matrices or simulated experimental systems did not necessarily establish their performance in formulated foods. Evaluation of the finished food is therefore necessary to determine whether the properties observed during ingredient characterization remain relevant after processing and incorporation with other components. Companion-animal feeding studies provide a separate application context for evaluating plant-derived ingredients in complete diets. Reported differences in apparent nutrient digestibility, fecal characteristics, fermentation metabolites, postprandial glycemic responses, and selected microbiota measurements depended on ingredient composition, inclusion level, processing, and dietary comparators. Changes in these outcomes were not uniformly associated with broader physiological benefits. Evidence concerning immune-related, metabolic, taurine-associated, and cardiac measurements remains limited by the experimental conditions and duration of the available studies. Direct comparison between dogs and cats is further constrained by differences in study coverage, dietary formulations, and measured endpoints. This review is limited by its selective narrative design, reliance on a single database, and screening restricted to the first 20 relevance-ranked records within each matrix-specific search. Relevant studies may therefore have been omitted, and the selected literature should not be regarded as a comprehensive evidence base. Differences in experimental systems, sample sizes, processing conditions, feeding durations, and outcome measurements also restrict comparisons across studies. In addition, related publications and experimental cohorts may not represent independent evidence.

Future investigations should report processing conditions, including temperature, moisture, residence time, screw configuration, and specific mechanical energy where applicable, together with ingredient inclusion levels on a dry-matter basis. Assessment of matrix properties before and after food manufacture should be combined with standardized digestibility and postprandial testing protocols. Longer feeding studies, independent replication, and clearly defined physiological endpoints are particularly needed when examining metabolic, microbiota-associated, immune-related, taurine, and cardiac outcomes. Evidence supporting physiological or health-related claims should be established in the finished food and the intended consumer or target species.

## Data Availability

No new data were created or analyzed in this study. Data sharing is not applicable to this article.

## Supplementary Materials

The following supporting information can be downloaded at: https://www.mdpi.com/article/10.3390/foods15213859/s1, Table S1: Study-level evidence for plant-derived matrices in companion-animal foods.
