# Supplementary material for: Plant-Derived Phytonutritional Ingredients for Food and Pet Food: Processing, Functionality, and Applications
Source: Foods. 2026 Oct 28;15(21):3859. doi: 10.3390/foods15213859 (PMC13763233; doi:10.3390/foods15213859)
Supplement: Supplementary file 1 [file foods-15-03859-s001.zip › foods-4599124-supplementary.pdf]

Supplementary Materials

Table S1. Study-level evidence for plant-derived matrices in companion-animal foods

Table S1.1. Plant-derived dietary fibers

Ingredient	Mode of incorporation/usage	Supplementary form	Inclusion and comparator	Species, sample and design	Reported findings / direction	Ref.
Guava fibre derived from dried guava peel and core residues	Incorporated into complete dry extruded dog diets at graded inclusion levels	Dry extruded dog food	– Control diet without added guava fibre<br>– Guava fibre inclusion at 3%, 6%, or 12% of diet (as-fed basis)<br>– Total dietary fibre concentrations: 10.75%, 13.13%, 16.63%, and 18.36% dry matter for control, 3%, 6%, and 12% guava fibre diets, respectively<br>– Guava fibre contained approximately 70.3% total dietary fibre with predominately insoluble fibre	24 healthy adult Beagle dogs; mean age 7 ± 2.2 years; mean body weight 12.4 ± 1.43 kg; completely randomized block design with four dietary treatments; two experimental blocks with six dogs per treatment; 15-day dietary adaptation followed by 6-day digestibility assessment; additional 6-day evaluation period for fecal fermentation products and gastrointestinal transit time; pair-housed during adaptation and individually housed during collection; twice-daily feeding with individual energy allowance adjusted to maintain body weight; palatability evaluated	– Reduced dietary metabolizable energy density<br>– Faster gastrointestinal transit time at 12% inclusion<br>– Maintained acceptable fecal quality across inclusion levels<br>– Reduced fecal acetate and propionate concentrations<br>– Supported acceptable diet palatability in adult dogs	[93]

Ingredient	Mode of incorporation/usage	Supplementary form	Inclusion and comparator	Species, sample and design	Reported findings / direction	Ref.
				by two-pan preference testing		
Powdered cellulose, granulated cellulose, and lignocellulose	Fiber sources mixed directly into complete wet diets prior to feeding	Commercial-style canned wet dog food	– Each fiber source included at 2% of diet (as-fed basis)<br>– Control diet without added fiber	8 adult Beagle dogs; replicated 4 × 4 Latin square design; 4 diets; 4 periods of 14 days each; daily feeding with fiber additive inclusion based on body weight	– Reduced gross energy digestibility relative to control diet<br>– Increased crude fiber digestibility with supplemental fiber inclusion<br>– Increased wet fecal output<br>– Maintained optimal fecal consistency and shape scores<br>– Preserved diet acceptance and voluntary food intake across fiber sources	[94]
Three baked diets with different cereal matrices: refined wheat, whole-grain rye, and a mixture of whole-grain rye and refined wheat	Refined wheat, whole-grain rye, or their equal mixture incorporated as the main carbohydrate source during formulation, then baked into complete diets	Complete baked dog diets	– Refined wheat diet: refined wheat provided 50% of total diet dry matter<br>– Whole-grain rye diet: whole-grain rye provided 50% of total diet dry matter<br>– Mixed diet: whole-grain rye 25% + refined wheat 25% of total diet dry matter (50% combined)	6 healthy male purpose-bred Beagle dogs; mean age 4.6 ± 0.95 years (range 1–7 years); mean body weight 14.6 ± 0.32 kg (range 13.6–15.7 kg); sequential feeding design with three experimental diet periods; refined wheat diet followed by randomized mixed rye/wheat and rye diets; 6-day acclimatization plus 15-day feeding per	– Increased fecal *Prevotella* abundance after whole grain rye consumption<br>– Modulated fecal microbiota composition according to carbohydrate source<br>– Tendency toward higher fecal acetic acid proportion with whole grain rye diet<br>– Reduced apparent total tract digestibility of crude protein, crude	[95]

Ingredient	Mode of incorporation/usage	Supplementary form	Inclusion and comparator	Species, sample and design	Reported findings / direction	Ref.
				period; 2–2.5 month washout with commercial diet between periods; indoor and outdoor group housing	fat, neutral detergent fiber, and gross energy with whole grain rye diet	
Miscanthus grass fiber, alone or blended with tomato pomace, compared with cellulose and beet pulp as dietary fiber sources	Fiber sources were incorporated into complete diet formulations before extrusion to produce dry kibble test diets	Dry extruded feline diet (complete kibble)	– Cellulose control diet: 7% cellulose<br>– Beet pulp diet: 9% beet pulp<br>– Miscanthus diet: 9% Miscanthus grass fiber<br>– Miscanthus plus tomato pomace diet: 7% Miscanthus grass fiber + 2% tomato pomace	28 healthy neutered adult domestic shorthair cats; randomized parallel-group feeding; 4 groups (n = 7/group); 7-day adaptation on the cellulose control diet; then 21-day feeding of assigned test diet; cats housed individually; water ad libitum; intake adjusted to maintain body weight	– Maintained food intake, fecal quality, and apparent total tract digestibility of macronutrients across diets<br>– Miscanthus diets produced fecal fermentation end-product patterns described as closer to cellulose than beet pulp<br>– Miscanthus diets were associated with higher fecal microbiota alpha diversity than the beet pulp diet in selected diversity metrics	[96]
• Miscanthus grass<br>• Traditional fibre sources: Cellulose, Beet pulp	Fibre ingredients were mixed into diets prior to extrusion	Dry extruded cat foods (Kibble)	*Miscanthus* grass at 10%, compared with cellulose- or beet pulp-containing diets.	12 adult American Shorthair cats; replicated Latin square design; dietary fiber sources (Miscanthus grass, cellulose, beet pulp); 9-day adaptation; 5-day total fecal	– Beet pulp had higher dry matter, organic matter, gross energy, and total dietary fibre digestibility than Miscanthus grass or cellulose<br>– Beet pulp produced softer stools and lower	[146]

Ingredient	Mode of incorporation/usage	Supplementary form	Inclusion and comparator	Species, sample and design	Reported findings / direction	Ref.
				collection; controlled twice-daily feeding	fecal dry matter than Miscanthus grass and cellulose<br>– Miscanthus grass had lower crude fat digestibility than cellulose and beet pulp in the digestibility trial<br>– In the hairball trial, Miscanthus grass did not change hair clump count, size, weight, or total hair clump weight<br>– Miscanthus grass reduced total hair weight per gram of dry feces and hair clumps per gram of dry feces compared with the control diet<br>– Miscanthus grass increased wet fecal output and produced firmer feces than the control hairball diet	
• Miscanthus grass<br>• Traditional fibre sources: cellulose and beet pulp	Fiber sources incorporated into complete diet formulations before extrusion to produce experimental dry diets	Dry extruded dog foods (Kibble)	*Miscanthus* grass at 10%, compared with cellulose- or beet pulp-containing diets.	12 adult Beagle dogs; individual housing; twice-daily feeding; replicated 3 × 3 Latin square design; three 14-day periods; diets: control, cellulose, beet	– Miscanthus grass and cellulose produced firmer stools than beet pulp<br>– Miscanthus grass showed fecal score and apparent nutrient	[97]

Ingredient	Mode of incorporation/usage	Supplementary form	Inclusion and comparator	Species, sample and design	Reported findings / direction	Ref.
				pulp, Miscanthus grass; 9-day adaptation	digestibility patterns similar to cellulose<br>– Beet pulp had higher apparent total-tract digestibility of dry matter, organic matter, gross energy, and total dietary fiber than Miscanthus grass and cellulose<br>– Crude protein digestibility was lower with beet pulp than with cellulose and Miscanthus grass<br>– Crude fat digestibility was lower with Miscanthus grass than with cellulose and beet pulp<br>– Wet fecal output differed among fiber sources, highest with cellulose, lowest with beet pulp, and intermediate with Miscanthus grass	
Sorghum fractions: whole sorghum, sorghum flour, and sorghum mill-feed enriched with bran and endosperm	Each sorghum fraction incorporated into the diet formulation before extrusion as the primary carbohydrate source	Dry extruded dog food	– Whole sorghum diet: whole sorghum 64.7%<br>– Sorghum flour diet: sorghum flour 63.11%	12 adult Beagle dogs; replicated 3 × 3 Latin square design; three dietary treatments; three 14-day periods	– Apparent total tract digestibility of dry matter, organic matter, crude protein, and gross energy differed	[28]

Ingredient	Mode of incorporation/usage	Supplementary form	Inclusion and comparator	Species, sample and design	Reported findings / direction	Ref.
			– Sorghum mill-feed diet: sorghum mill-feed 67.6%		among sorghum fractions<br>– Total dietary fiber digestibility varied by fraction type, with higher fiber digestibility observed in sorghum flour compared with whole sorghum and mill feed.<br>– Plasma and fecal antioxidant capacity differed among dietary treatments.<br>– Fecal output and fecal score remained within physiologically acceptable ranges, with no evidence of adverse stool quality	
Fine cassava fibre	Incorporated into the diet formulation before extrusion as a fiber source replacing conventional fiber ingredients	Dry extruded dog food	– Control diet without added cassava fibre (4.31% total dietary fibre, dry matter basis)<br>– Cassava fibre diet containing 9.58% cassava fibre (as-fed basis)	8 adult Beagle dogs; replicated 4 × 4 Latin square design; four dietary treatments; four 14-day periods	– Cassava fibre diet showed higher palatability than the control diet based on first choice and intake ratio<br>– Cassava fibre did not alter most apparent total-tract nutrient digestibility coefficients or metabolizable energy, but increased total, insoluble, and	[105]

Ingredient	Mode of incorporation/usage	Supplementary form	Inclusion and comparator	Species, sample and design	Reported findings / direction	Ref.
					soluble dietary fibre digestibility<br>– Cassava fibre increased fecal acetate, butyrate, and total short-chain fatty acids and reduced fecal phenol, indole, and isobutyrate concentrations<br>– Fecal microbiota composition shifted with higher relative abundance of selected genera including *Blautia*, *Faecalibacterium*, and *Fusobacterium*	
Cassava fiber compared with conventional dietary fiber sources, including cellulose, beet pulp, and lignocellulose	Incorporated into the diet formulation before extrusion as the primary fiber source	Dry extruded dog food	– Control diet without added fiber source<br>– Cassava fiber included at 40, 80, or 120 g/kg diet<br>– Cassava fiber diets provided total dietary fiber concentrations of 61, 72, and 83 g/kg dry matter, respectively	8 adult Beagle dogs; replicated Latin square design; three dietary treatments; 14-day experimental periods	– Apparent total-tract digestibility of dry matter, organic matter, crude protein, gross energy, and dietary fiber differed among fiber sources<br>– Fecal output, fecal dry matter, and fecal score varied according to fiber type<br>– Fecal acetate, propionate, butyrate, and total short-chain fatty acid	[129]

Ingredient	Mode of incorporation/usage	Supplementary form	Inclusion and comparator	Species, sample and design	Reported findings / direction	Ref.
					concentrations differed among treatments<br>– Fecal branched-chain fatty acids and protein-fermentation metabolites differed among fiber sources<br>– Fecal microbiota composition shifted according to fiber source, with differences in relative abundance of selected bacterial taxa	
Soluble corn fiber	Incorporated directly into the diet formulation before extrusion as a supplemental fermentable fiber source	Dry extruded dog food	– Control diet without added soluble corn fiber<br>– Soluble corn fiber included at 0.1%, 0.5%, or 1.0% of diet (w/w)	20 adult dogs; randomized design; 4 dietary treatments; 21-day feeding period; control vs graded soluble corn fiber inclusion; daily feeding	– Improved fecal score at 1% inclusion<br>– Increased fecal acetate, propionate, butyrate, and total short-chain fatty acids<br>– Altered fecal microbiota diversity and composition<br>– Maintained overall dietary tolerance during short-term feeding	[100]
Wheat dextrin soluble fiber	Mixed with the regular diet once daily as a powder supplement	Oral supplementation alongside habitual diet	– Wheat dextrin administered once daily with gradual increase to maximum dose<br>– Dogs <20 kg: up to 4.5 g/day	17 dogs with chronic enteropathy; randomized single-blinded parallel design; wheat dextrin vs maltodextrin placebo; 7-day baseline	– Maintained dietary acceptance and intake during supplementation<br>– Changes in fecal score reported in a subset of dogs	[104]

Ingredient	Mode of incorporation/usage	Supplementary form	Inclusion and comparator	Species, sample and design	Reported findings / direction	Ref.
			– Dogs ≥20 kg: up to 9 g/day<br>– Median intake: ~0.4 g/kg/day (range ~0.2–0.9 g/kg/day)	observation followed by 28-day supplementation; gradual dose escalation over 14 days	– Limited or inconsistent changes in fecal short-chain fatty acids<br>– No consistent shifts in fecal microbial richness or diversity indices	
Powdered cellulose	Cellulose powder distributed equally over the daily ration as an oral dietary fiber supplement	Wet commercial dog food	– Cellulose dose: 0.5 g/kg body weight per day for 7 days<br>– Control group: crushed hydrolyzed kibble powder with no cellulose<br>– Both groups received a commercial limited-ingredient wet diet for 10 days	19 client-owned dogs with uncomplicated acute diarrhea; prospective randomized double-blinded clinical trial; cellulose group (n = 10) vs control group (n = 9); dogs ≥9 months old; diarrhea duration ≤5 days before presentation; 7-day test powder administration; 21-day owner diary monitoring	– Faster improvement in stool consistency during early recovery<br>– Clinical recovery within 1–6 days<br>– Improved fecal quality during acute diarrhea management<br>– Recovery of core intestinal microbiota during follow-up<br>– Reduced diarrhea severity scores during feeding period	[143]
Three dietary fibre supplements: psyllium husk, green banana flour resistant starch, and methylcellulose	Each fiber supplement was administered orally with the regular food ration during sequential crossover supplementation periods	Commercial complete canine diet supplemented with individual dietary fiber preparations	– Dogs < 5 kg: 2 g supplement twice daily<br>– Dogs 5–10 kg: 4 g supplement twice daily<br>– Dogs > 10–30 kg: 8 g supplement twice daily<br>– Dogs > 30–50 kg: 12 g supplement twice daily<br>– Dogs > 50 kg: 16 g supplement twice daily	17 healthy adult dogs; randomized crossover trial; transition to standardized complete diet before supplementation; sequential psyllium, resistant starch, and methylcellulose supplementation	– Increased fecal firmness with methylcellulose supplementation<br>– Altered fecal microbiota richness after resistant starch supplementation<br>– Reduced abundance of several SCFA-producing bacterial	[130]

Ingredient	Mode of incorporation/usage	Supplementary form	Inclusion and comparator	Species, sample and design	Reported findings / direction	Ref.
			– Separate supplementation periods for psyllium husk, resistant starch (green banana flour), and methylcellulose	periods; washout phase between treatments	genera with resistant starch<br>– Increased abundance of selected *Bacteroidota*-, *Pseudomonadota*-, and *Actinomycetota*-associated taxa<br>– Minor taxonomic shifts following psyllium supplementation<br>– Fiber-specific modulation of intestinal microbial composition	
Citrus pulp pellets and orange fiber	Incorporated into the diet formulation before extrusion by replacing maize at specified inclusion levels	Dry extruded adult dog food	– Control diet: no added citrus fiber source<br>– Citrus pulp pellet: 30 g/kg, 60 g/kg, or 120 g/kg diet as-fed<br>– Orange fiber: 60 g/kg diet as-fed	30 adult Beagle dogs; randomized block design; 5 dietary treatments; 6 dogs/treatment; control vs 3% citrus pulp pellet, 6% citrus pulp pellet, 12% citrus pulp pellet, or 6% orange fiber; 15-day digestibility trial; 10-day diet adaptation; 5-day total fecal collection; individualized feeding to maintenance energy requirement; palatability test with 37 dogs; two-pan method;	– Increased fecal total SCFAs<br>– Increased fecal acetate and butyrate<br>– Increased fecal lactate<br>– Lower fecal pH<br>– Increased total dietary fiber digestibility<br>– Higher intake preference for orange-fiber diet<br>– Maintained diet acceptance without vomiting or diarrhea	[99]

Ingredient	Mode of incorporation/usage	Supplementary form	Inclusion and comparator	Species, sample and design	Reported findings / direction	Ref.
				first choice and intake ratio comparisons		
Orange fiber derived from citrus-processing coproducts	Incorporated into the diet formulation before extrusion as a dietary fiber source replacing maize starch	Dry extruded dog food	– Control diet without orange fiber<br>– Orange fiber inclusion: 3%, 6%, or 12% of diet as-fed basis	8 healthy adult Beagle dogs; replicated 4 × 4 Latin square design; control vs diets containing 3%, 6%, or 12% orange fiber; four experimental periods	– Increased fecal acetate and total SCFAs<br>– Lower fecal pH<br>– Increased fecal output and fecal moisture<br>– Reduced mean retention time of digesta<br>– Increased dietary fiber digestibility<br>– Maintained acceptable stool consistency during feeding trial	[103]
Orange fiber derived from citrus-processing coproducts	Incorporated into the diet formulation before extrusion as a dietary fiber source replacing maize starch	Dry extruded kibble diet for adult cats	– Control diet without orange fiber<br>– Orange fiber inclusion: 3%, 6%, or 12% of diet as-fed basis	8 healthy adult cats; replicated 4 × 4 Latin square design; control vs diets containing 3%, 6%, or 12% orange fiber; four experimental periods	– Increased fecal acetate concentration<br>– Increased total fecal SCFAs<br>– Lower fecal pH<br>– Increased fecal bulk and fecal moisture<br>– Reduced gastrointestinal mean retention time<br>– Increased total dietary fiber digestibility<br>– Maintained acceptable fecal quality during feeding period	[145]
Dried apple pomace	Included directly into the diet formulation	Dry extruded dog food	– Control diet without apple pomace	8 healthy adult Beagle dogs; replicated 4 × 4 Latin square design;	– Increased fecal butyrate concentration	[102]

Ingredient	Mode of incorporation/usage	Supplementary form	Inclusion and comparator	Species, sample and design	Reported findings / direction	Ref.
	before extrusion as a dietary fiber source		– Apple pomace inclusion: 3%, 6%, or 12% of diet as-fed basis	control vs diets containing 3%, 6%, or 12% dried apple pomace; four experimental periods	– Increased total fecal SCFAs<br>– Lower fecal pH<br>– Increased abundance of *Faecalibacterium* spp.<br>– Increased abundance of *Lachnospira* spp.<br>– Increased fecal bulk and fecal moisture<br>– Increased total dietary fiber digestibility	
Fiber bundle composed of ground pecan shells, dried pelleted beet pulp, ground citrus pulp, whole brown flax seed, and cranberry pomace	Mixed directly into the diet at graded inclusion levels through extrusion as a replacement for cellulose	Dry extruded grain-rich adult dog food	– Control diet without fiber bundle<br>– Fiber bundle inclusion: 1%, 2%, or 4% w/w on a dry matter basis<br>– Fiber bundle replaced all or part of cellulose in the control formulation	48 healthy adult dogs (24 females, 24 males); randomized parallel-group design; 28-day pre-feed control period; 31-day feeding trial; control vs diets containing 1%, 2%, or 4% fiber bundle; 12 dogs/group; pair-housed; individualized feeding based on caloric requirements	– Increased fecal butyric acid, valeric acid, and hexanoic acid at 4% inclusion<br>– Increased fecal hesperidin, hesperetin, and ponciretin<br>– Increased fecal secoisolariciresinol diglucoside, secoisolariciresinol, and enterodiol<br>– Increased fecal arabinose at 4% inclusion<br>– Increased fecal ribulose/xylulose at 4% inclusion on day 10<br>– Increased fiber-bound polyphenol-derived fecal metabolites	[101]

Ingredient	Mode of incorporation/usage	Supplementary form	Inclusion and comparator	Species, sample and design	Reported findings / direction	Ref.
Red ginseng dietary fiber	Mixed into the diet as a supplemental dietary fiber source at graded intake levels	Commercial dog diet	– High-dose intake: 8 g/5 kg body weight/day (n = 16)<br>– Low-dose intake: 3 g/5 kg body weight/day (n = 12)<br>– Control group without red ginseng dietary fiber supplementation (n = 12)	40 healthy companion dogs of various breeds; mean age 4.8 ± 2.6 years; mean body weight 8.3 ± 5.2 kg; randomized parallel-group design; control vs low-dose (3 g/5 kg BW/day) or high-dose (8 g/5 kg BW/day) red ginseng dietary fiber supplementation; 8-week feeding period; dogs maintained on habitual diets with added supplementation	– Increased fecal acetate and propionate concentrations<br>– Increased abundance of *Sarcina* spp. and *Proteiniclasticum* spp.<br>– Reduced abundance of *Helicobacter* spp.<br>– Increased serum IgA concentration<br>– Increased serum IgM concentration<br>– Improved fecal microbial diversity and community structure	[26]

Table S1.2. Plant-derived dietary fibers

Ingredient	Mode of incorporation/usage	Supplementary form	Inclusion and comparator	Species, sample and design	Reported findings / direction	Ref.
Corn-based resistant-starch kibble produced by high-, medium-, or low-shear extrusion	Resistant starch levels were modified through extrusion shear processing conditions during kibble manufacture	Dry extruded dog kibble	– Whole yellow corn included at 65% of the formula<br>– High-shear extruded kibble: resistant starch 0.52%<br>– Medium-shear extruded kibble: resistant starch 1.85%<br>– Low-shear extruded kibble: resistant starch 5.01%<br>– All diets formulated to similar macronutrient composition	12 healthy adult female Beagles; mean age 3.5 ± 0.7 years; mean body weight 10.0 ± 0.8 kg; replicated 3 × 3 Latin square design; high-, medium-, and low-shear extruded diets fed across three experimental periods; 28-day feeding periods with 22-day adaptation and 5-day fecal collection; individually housed and fed to maintenance energy requirements	– Increased fecal butyric acid concentration with low-shear diet<br>– Increased fecal oligosaccharides and saccharide-derived metabolites<br>– Increased fecal arabinose, ribulose/xylulose, and glucose concentrations<br>– Increased fecal fecalibacteria abundance with low-shear diet<br>– Increased fecal saccharolytic fermentation by-products<br>– Reduced fecal pH with resistant starch-rich diets	[118]
Brewer's rice and whole grain corn	Brewer's rice and whole grain corn with varying resistant starch preservation through extrusion processing	Dry extruded grain-based cat food	– Low-resistant-starch food: high-shear extrusion; non-gelatinized starch used as putative resistant starch, 0.5%<br>– High-resistant-starch food: low-shear extrusion; non-gelatinized starch used as putative resistant starch, 7.9%	40 healthy adult domestic shorthair cats; spayed or neutered; mean age 8.9 ± 0.5 years; randomized 7-week longitudinal feeding trial; high-shear low-resistant-starch food (n = 17) vs low-shear high-resistant-starch food (n = 19); individually housed; fed to maintain body weight; water ad libitum; fecal collections after 3 and 6 weeks of feeding;	– Increased fecal butyric acid concentration<br>– Increased fecal straight-chain-to-branched-chain fatty acid ratio<br>– Reduced fecal ammonia concentration after 6 weeks<br>– Increased fecal immunoglobulin A concentration<br>– Increased fecal microbial alpha diversity	[120]

Ingredient	Mode of incorporation/usage	Supplementary form	Inclusion and comparator	Species, sample and design	Reported findings / direction	Ref.
					– Increased fecal Lactobacillus abundance<br>– Increased fecal saccharolytic metabolite profile<br>– Improved stool firmness	
Corn-based resistant-starch and slowly digestible starch matrices generated by pelleting, baking, or extrusion	The same corn-based formula was processed by pelleting, baking, or extrusion to create different starch-cooking intensities and different levels of resistant starch, slowly digestible starch, and rapidly digestible starch	Pelleted, baked, or extruded corn-based dog food	– Corn inclusion: 56% of diet formulation as sole starch source<br>– Pelleted diet: lowest starch cooks and highest resistant starch<br>– Baked diet: intermediate starch cooks and resistant starch<br>– Extruded diet: highest starch cooks and lowest resistant starch<br>– Baked dextrose-control diet: corn replaced with dextrose	12 adult Beagle dogs (4 spayed females, 8 neutered males); mean age 6.5 ± 0.23 years; mean body weight 11.6 ± 1.8 kg; replicated 4 × 4 Latin square design; pelleted, baked, extruded corn-based diets, and baked dextrose-control diet; 14-day experimental periods with 9-day adaptation and 5-day fecal collection; individually housed	– Improved fecal quality with extruded diet compared with baked and pelleted diets<br>– Increased apparent total tract digestibility with extruded diet<br>– Increased fecal butyrate tendency with extruded diet<br>– Maintained similar fecal SCFA concentrations among corn-based diets<br>– Lower fecal pH in corn-based diets compared with dextrose control<br>– Reduced branched-chain fatty acid proportions in corn-based diets<br>– Maintained similar fecal microbial alpha diversity among corn-based diets	[133]
High-amylose Dodamssal rice, compared with normal-amylose rice	High-amylose resistant-starch rice was incorporated into high-fat or normal-fat diet	Cooked rice-based experimental dog diets with normal- or high-fat formulations	– Experiment 1: normal-fat basal diet (control)<br>– High-fat diet with 12% normal-amylose rice	– Experiment 1: 24 spayed female small-breed dogs; mean age 7.60 ± 0.01 years; initial body weight 4.73 ± 0.31 kg; 9 Poodles, 6	– Reduced body weight gain with high-amylose resistant-starch rice diet	[134]

Ingredient	Mode of incorporation/usage	Supplementary form	Inclusion and comparator	Species, sample and design	Reported findings / direction	Ref.
	formulations as a carbohydrate source		– High-fat diet with 12% high-amylose resistant-starch rice (Dodamssal rice)<br>– Experiment 2: normal-fat diet with normal-amylose rice (NAR) or high-amylose resistant-starch rice (HAR)	Maltese, and 9 Miniature Schnauzers; randomized into control, high-fat normal-amylose rice, and high-fat high-amylose resistant-starch rice groups; 24-week feeding period<br>– Experiment 2: 8 spayed female small-breed dogs; mean age 9.60 ± 0.01 years; initial body weight 4.28 ± 0.63 kg; 2 Schnauzers, 2 Maltese, 2 Yorkshire Terriers, 1 Shih Tzu, and 1 Pomeranian; randomized into normal-amylose rice and high-amylose resistant-starch rice groups; 4-week feeding period	– Lower apparent metabolizable energy intake with high-amylose rice<br>– Reduced nutrient digestibility associated with resistant starch intake<br>– Lower serum alanine aminotransferase concentration compared with high-fat normal-amylose rice diet<br>– Reduced body weight after 4 weeks in dogs fed high-amylose resistant-starch rice<br>– Maintained normal hematological and biochemical safety profiles during feeding period	
Corn resistant starch produced by heating–cooling treatment, compared with rice-based starch	Corn resistant starch was incorporated into the diet formulation as the primary carbohydrate source replacing rice	Cooked experimental dog diets composed of rice or corn resistant starch with chicken breast meal	– Control diet: rice powder 31.9% and chicken breast meal 15.0%<br>– Corn resistant starch diet: heat–cool-treated corn starch powder 29.2% and chicken breast meal 17.7%<br>– Resistant starch content of ingredients: rice 2.33%; heat–cool-treated corn starch 6.69%<br>– Resistant starch content of experimental diets:	10 adult Beagle dogs (4 spayed females, 6 castrated males); mean age 2.9 ± 0.05 years; randomized parallel-group design; control group (n = 5) fed rice-based diet vs treatment group (n = 5) fed corn resistant-starch diet; 16-week feeding period; individually housed under controlled temperature and humidity; fed twice daily; body weight monitored biweekly; chromium oxide (0.5%)	– Reduced body weight gain compared with control diet<br>– Reduced apparent total tract digestibility of dry matter<br>– Reduced apparent total tract digestibility of nitrogen-free extract<br>– Reduced apparent total tract digestibility of organic matter<br>– Increased serum adiponectin concentration	[135]

Ingredient	Mode of incorporation/usage	Supplementary form	Inclusion and comparator	Species, sample and design	Reported findings / direction	Ref.
			control diet 1.09%; corn resistant starch diet 3.12%<br>– Corn starch processed by steam heating above 100 °C for 30 min followed by cooling at 4 °C for 24 h	added during final week for digestibility assessment; blood sampling after 16-h fasting at baseline and week 16; nutrient digestibility, body weight change, and serum metabolic parameters evaluated	– Maintained normal hematological and biochemical parameters within reference range	
Flours of round pea, lentil, faba bean, wrinkled pea, and rice (control)	Pulse flour was incorporated into complete pet food formulations and processed through extrusion under controlled extrusion conditions	Dry extruded pulse-based pet foods coated with chicken fat and canola oil	– Diets formulated to contain similar macronutrient levels: starch (~20%, db), protein (~26%, db), fiber (~15%, db), and fat (~15%, db)<br>– Rice or pulse flours served as the sole metabolizable carbohydrate source in each diet<br>– Two extrusion processing conditions applied: mild processing (C1) and extreme processing (C2)<br>– C1 condition used low-shear screw configuration<br>– C2 condition used high-shear screw configuration<br>– Extrudates air-dried at room temperature for 24 h before storage at 4 °C<br>– *In vivo* Beagle feeding trial performed using diets processed under C1 condition	8 adult Beagle dogs (4 neutered males, 4 spayed females); body weight 8.87 ± 0.90 kg; 2–4 years old; randomized crossover blinded repeated feeding trial; extruded diets containing round pea, lentil, faba bean, wrinkled pea, or rice flour fed continuously for 7 days; 3-day washout period with commercial grain-containing diet between treatments	– High total tract apparent starch digestibility in all extruded diets<br>– Lower starch digestibility in wrinkled pea diet compared with rice control<br>– Pulse crop type influenced resistant starch and slowly digestible starch fractions after extrusion<br>– Extrusion processing altered starch physicochemical properties among pulse flours<br>– Differences in starch gelatinization and starch accessibility among pulse-based diets	[137]

Ingredient	Mode of incorporation/usage	Supplementary form	Inclusion and comparator	Species, sample and design	Reported findings / direction	Ref.
Potato flour–derived resistant starch; oat groats, beet pulp, pea fiber, and inulin-based fiber blend	Resistant starch, dietary fibers, prebiotic, probiotic, and immune-modulating ingredients were incorporated into complete extruded feline kibble as diet-level gut microbiota modulators	Dry extruded cat food	– Resistant starch diet (ERS): potato flour (21.0%) as the primary resistant starch source; brewers rice (20.58%); salmon slurry (20.0%); chicken meal (20.0%)<br>– Fiber, prebiotic, probiotic blend diet (FPPB): oat groats (20.0%), dried green peas (12.91%), dried plain beet pulp (3.0%), flaxseed meal (3.0%), pea fiber (3.0%), inulin (0.10%), probiotic blend (2.30%) containing *Lactobacillus acidophilus* and *Enterococcus faecium*<br>– Fiber, prebiotic, probiotic blend plus immune-modulating ingredients diet (iFPPB): oat groats (16.66%), dried green peas (12.0%), dried plain beet pulp (4.0%), flaxseed meal (3.0%), pea fiber (3.0%), spray-dried plasma (3.0%), probiotic blend (2.30%), and yeast fermentation product (0.25%)<br>– All diets formulated to meet AAFCO nutrient recommendations for adult cats at maintenance	12 healthy adult female domestic shorthair cats; mean age 9.6 ± 4.0 years; mean body weight 3.9 ± 1.0 kg; replicated 3 × 3 Latin square design; cats fed resistant starch-enriched diet (ERS), fiber-prebiotic-probiotic blend diet (FPPB), or fiber-prebiotic-probiotic blend plus immunomodulator diet (iFPPB); 28-day experimental periods with 22-day adaptation, fecal collection on days 23–27, and blood collection on day 28	– Increased fecal butyrate concentration in cats fed resistant starch diet<br>– Reduced fecal propionate concentration with resistant starch diet<br>– Reduced fecal branched-chain fatty acids, phenols, indoles, and ammonia with resistant starch diet<br>– Reduced fecal pH in cats fed resistant starch diet<br>– Increased fecal microbial alpha diversity with fiber-enriched diets<br>– Increased relative abundance of Firmicutes and Bacteroidetes with fiber, prebiotic, probiotic blend diets<br>– Increased relative abundance of Actinobacteria with resistant starch diet<br>– Reduced serum cholesterol concentration in cats fed resistant starch diet	[119]
Corn, brown rice, sorghum, potato starch,	Each starch source was incorporated into complete	Dry extruded dog food	– Diets formulated to contain similar starch	14 healthy adult Beagle dogs; randomized block	– Higher apparent total tract digestibility of	[136]

Ingredient	Mode of incorporation/usage	Supplementary form	Inclusion and comparator	Species, sample and design	Reported findings / direction	Ref.
sweet potato flour, chickpea, and pea flour as dietary starch sources	canine diet formulations before extrusion as the primary carbohydrate source		concentrations (300 g/kg diet)<br>– Corn, brown rice, sorghum, potato starch, sweet potato flour, chickpea, or pea flour used as the primary starch source in each formulation<br>– Diets formulated to meet maintenance nutrient requirements for adult dogs according to FEDIAF guidelines<br>– Experimental diets processed under standardized extrusion conditions	design with three experimental periods; dogs fed extruded diets containing corn, brown rice, sorghum, potato starch, sweet potato flour, chickpea, or pea flour; 15-day feeding periods; six repetitions per treatment; individually housed and fed to maintenance energy requirements	nutrients in brown rice and pea diets<br>– Lower apparent total tract digestibility in sweet potato diet<br>– Higher metabolizable energy content in corn and brown rice diets<br>– Reduced postprandial glycemic response with chickpea and pea diets<br>– Increased fecal acetate and total short-chain fatty acids with pea diet<br>– Reduced fecal branched-chain fatty acids and ammonia with pea diet<br>– Distinct fecal microbial and fermentative metabolite profiles among starch sources	
Amaranth, white proso millet, quinoa, and oat groats compared with rice as ancient grain carbohydrate sources	Each ancient grain was incorporated into complete canine diet formulations before extrusion as the primary carbohydrate source	Dry extruded dog food	– Control diet: rice flour 40.2% as the main carbohydrate source<br>– Amaranth diet: amaranth 40.0% plus rice flour 10.0%<br>– White proso millet diet: white proso millet 40.0% plus rice flour 9.8%<br>– Quinoa diet: quinoa 40.0% plus rice flour 8.3%	10 adult intact female Beagle dogs; mean age 4.2 ± 1.14 years; mean body weight 11.1 ± 1.17 kg; mean body condition score 5.7 ± 0.7; replicated 5 × 5 Latin square design; dogs fed extruded diets containing rice, amaranth, white proso millet, quinoa, or oat groats; individually housed during adaptation and placed in metabolic cages during collection	– Higher dry matter and organic matter digestibility with white proso millet diet<br>– Higher total dietary fiber digestibility with white proso millet diet<br>– Increased fecal total short-chain fatty acids with amaranth and oat groats diets<br>– Increased fecal propionate and butyrate	[139]

Ingredient	Mode of incorporation/usage	Supplementary form	Inclusion and comparator	Species, sample and design	Reported findings / direction	Ref.
			– Oat groats diet: oat groats 40.1% plus rice flour 10.0%	phases; fed twice daily to maintain body weight with free access to water	concentrations with amaranth and oat groats diets<br>– Increased relative abundance of *Lachnospiraceae* with oat groats diet<br>– Reduced relative abundance of *Fusobacteriaceae* with amaranth and oat groats diets<br>– Maintained acceptable stool quality across dietary treatments	
Potato flour, cassava starch, dried sweet potato, rice, and wheat as dietary carbohydrate sources	Each carbohydrate source was incorporated into complete feline diet formulations as the sole starch source before extrusion processing	Dry extruded cat foods	– Potato diet: potato flour 22.8% plus chicken powder 30.0%<br>– Cassava diet: cassava starch 19.7% plus chicken powder 30.0%<br>– Sweet potato diet: dried sweet potato 32.0% plus chicken powder 30.0%<br>– Rice diet: rice 24.7% plus chicken powder 30.0%<br>– Wheat diet: wheat 28.9% plus chicken powder 30.0%<br>– Each diet contained one carbohydrate source as the sole starch source<br>– Experimental diets formulated to provide 4200 kcal ME/kg and meet	30 young cats (16 females, 14 males); mean age 0.60 ± 0.02 years; mean body weight 3.14 ± 0.18 kg; including 6 American Shorthairs, 5 Siamese, 14 British Shorthairs, and 5 Ragdolls; randomized into five dietary groups based on breed, body weight, and sex; cats fed potato-, cassava-, sweet potato-, rice-, or wheat-based diets; 34-day study with 7-day dietary adaptation and 28-day feeding trial; individually housed during formal experiment	– Lower postprandial glucose response with cassava diet<br>– Lower mean glucose concentration and glucose AUC values with cassava diet<br>– Higher gut microbial diversity with cassava and sweet potato diets<br>– Increased relative abundance of *Fusobacterium*, *Veillonella*, and *Actinobacillus* with sweet potato diet<br>– Increased relative abundance of *Delftia*, *Shinella*, *Rothia*, and	[141]

Ingredient	Mode of incorporation/usage	Supplementary form	Inclusion and comparator	Species, sample and design	Reported findings / direction	Ref.
			AAFCO nutrient recommendations for young cats		*Hydrogenophaga* with cassava diet<br>– Maintained normal growth performance and stool quality across dietary treatments<br>– Lower circulating triglyceride, total cholesterol, HDL, and LDL concentrations in cats fed cassava diet	
Traditional grains (corn and wheat), whole grains (oats and rye), grain-free legumes (peas and lentils), and vegan diet matrices	Commercial extruded diets containing different starch-source compositions were fed directly as complete diets to evaluate gastric emptying responses	Dry extruded dog food	– Traditional diet: total starch 41.69 ± 1.48% DM; resistant starch 0.41 ± 0.02% DM<br>– Whole-grain diet: total starch 40.83 ± 1.90% DM; resistant starch 0.16 ± 0.01% DM<br>– Grain-free diet: total starch 33.91 ± 0.44% DM; resistant starch 0.56 ± 0.02% DM<br>– Vegan diet: total starch 48.06 ± 4.26% DM; resistant starch 0.26 ± 0.03% DM<br>– Commercial extruded diets differed in starch source composition, available carbohydrate, and resistant starch concentrations	11 healthy adult Siberian Huskies; randomized partially replicated 6 × 6 Latin square design; dogs received traditional, grain-free, whole-grain, and vegan commercial extruded diets once each and glucose control twice; acetaminophen administered orally with each meal as a gastric emptying marker	– Faster gastric emptying rate with grain-free diet<br>– Greater acetaminophen peak concentration and area under the curve with grain-free diet<br>– Delayed gastric emptying response with whole-grain diet<br>– Dietary starch composition influenced postprandial gastric emptying kinetics<br>– Resistant starch and available carbohydrate contents were associated with differences in gastric transit dynamics	[138]
Rice- or potato-based starch fractions	Rice- or potato-based complementary foods were	Raw meat-based wet dog diet	– Control diet: homemade diet containing boiled rice	30 healthy shelter dogs initially enrolled; final	– Lower fecal ammonia nitrogen concentration	[140]

Ingredient	Mode of incorporation/usage	Supplementary form	Inclusion and comparator	Species, sample and design	Reported findings / direction	Ref.
	mixed with raw beef meat and water to prepare wet raw meat-based meals, replacing the shelter's usual rice-and-pasta homemade diet in two treatment groups		and boiled wheat pasta (1:1 ratio) with fresh raw beef meat<br>– Rice starch diet (B1): ~70% raw beef meat plus ~30% rice-based complementary food containing rice flour, chickpea flour, oat flakes, carrots, algae-derived omega-3 fatty acids, and vitamin–mineral premix<br>– Potato starch diet (B2): ~70% raw beef meat plus ~30% potato-based complementary food containing potato, chickpea flour, beet pulp, carrots, algae-derived omega-3 fatty acids, and vitamin–mineral premix<br>– Rice, chickpeas, and potato ingredients were autoclaved, air-dried, and milled before diet preparation<br>– Diets formulated to meet NRC nutrient recommendations for adult dogs at maintenance	groups included 8 dogs fed control diet, 10 dogs fed rice-based starch diet, and 9 dogs fed potato-based starch diet due to adoptions during the trial; groups balanced for age, sex, and body weight; dogs housed individually overnight and allowed free movement in recreational park during daytime; 15-day feeding trial following 90-day pre-feeding period with homemade rice–pasta–raw meat diet	with potato-based starch diet<br>– Lower fecal pH with potato-based starch diet<br>– Increased fecal lactic acid proportion with potato-based starch diet<br>– Increased relative abundance of SMB53 genus with rice-based starch diet<br>– Increased relative abundance of Megamonas genus with rice- and potato-based starch diets<br>– Distinct modulation of fecal microbial populations according to starch source and resistant starch characteristics	
High-amylose maize resistant starch (Hi-maize 260)	Resistant starch, amylopectin cornstarch, and cellulose blends were mixed with water and top-dressed onto the basal diet before feeding	Dry extruded dog food with resistant starch top dressing	– Basal diet contained 6.25% resistant starch on a dry matter basis<br>– Additional resistant starch treatments: 0%, 1%,	7 healthy intact adult female hound-cross dogs; mean age 5.3 years; body weight 20–25 kg; incomplete 5 × 5 Latin square design with five	– Increased fecal *Faecalibacterium* abundance with resistant starch supplementation	[128]

Ingredient	Mode of incorporation/usage	Supplementary form	Inclusion and comparator	Species, sample and design	Reported findings / direction	Ref.
			2%, 3%, or 4% Hi-maize 260 supplementation<br>– Hi-maize 260 consisted of ~60% resistant starch and ~40% digestible starch<br>– Daily resistant starch intake: 18.3 g/day (0%), 20.1 g/day (1%), 21.9 g/day (2%), 23.7 g/day (3%), and 25.5 g/day (4%)<br>– Dogs received 300 g/day of the basal extruded diet with starch blends formulated to remain iso-energetic	dietary treatments and five 21-day periods; diets included graded resistant starch concentrations (0–4%); 17-day dietary adaptation followed by 4-day fecal collection; dogs individually housed in temperature-controlled rooms with social enrichment	– Reduced fecal pH with increasing resistant starch intake<br>– Increased fecal output with graded resistant starch concentrations<br>– Altered apparent total tract digestibility of dry matter, organic matter, crude protein, fat, and gross energy<br>– Maintained normal fecal consistency across resistant starch treatments	

Table S1. 3. Prebiotic fibers and oligosaccharide-containing ingredients

Ingredient	Mode of incorporation/usage	Supplementary form	Inclusion and comparator	Species, sample and design	Reported findings / direction	Ref.
Chicory root powder as a source of fermentable prebiotic fiber and inulin-type fructans	Chicory root powder was incorporated directly into the diet formulation before extrusion at graded inclusion levels	Dry extruded dog food	– Control diet: basal diet without chicory root powder<br>– Low-dose chicory group: 5 g/kg feed dry matter<br>– Medium-dose chicory group: 10 g/kg feed dry matter<br>– High-dose chicory group: 15 g/kg feed dry matter	16 Labrador puppies; age 5–6 months; average body weight 6.64 ± 0.40 kg; completely randomized design; four dietary groups with four puppies per group; groups included control, 5 g/kg chicory, 10 g/kg chicory, and 15 g/kg chicory diets; total study duration 65 days; experimental feeding conducted for 45 days before a 4-day digestion trial	– Increased fecal acetate, propionate, butyrate, lactate, and total short-chain fatty acid concentrations with chicory supplementation<br>– Reduced fecal pH with increasing chicory inclusion<br>– Increased fecal Lactobacillus and Bifidobacterium counts<br>– Reduced fecal Clostridium and coliform populations at higher chicory inclusion levels<br>– Increased calcium digestibility at 10 and 15 g/kg supplementation<br>– Reduced plasma glucose, triglycerides, total cholesterol, LDL cholesterol, VLDL cholesterol, and blood urea nitrogen in selected chicory groups<br>– Enhanced delayed-type hypersensitivity response and increased plasma immunoglobulin G following chicory supplementation	[122]

Ingredient	Mode of incorporation/usage	Supplementary form	Inclusion and comparator	Species, sample and design	Reported findings / direction	Ref.
Chicory root powder included as a source of fermentable prebiotic fiber in wheat- and sorghum-based diets	Chicory root powder was incorporated directly into cereal-based diet formulations before pressure cooking	Pressure-cooked dog diets based on wheat or sorghum cereal matrices	– Wheat-based control diet: no chicory root powder<br>– Wheat-based prebiotic diet: chicory root powder at 10 g/kg diet<br>– Sorghum-based control diet: no chicory root powder<br>– Sorghum-based prebiotic diet: chicory root powder at 10 g/kg diet	16 healthy adult female Labrador dogs; approximately 2 years old; mean body weight 20 ± 0.1 kg; randomized into four equal dietary groups; 9-week feeding experiment; cereal sources included wheat and sorghum with or without chicory supplementation; dogs individually housed with free access to water and daily exercise/socialization	– Reduced fecal ammonia concentration in wheat-based diet supplemented with chicory<br>– Increased fecal Lactobacillus populations with chicory supplementation<br>– Increased fecal Bifidobacterium populations with chicory supplementation tendency<br>– Reduced fecal coliform counts in chicory-supplemented diets<br>– Enhanced delayed-type hypersensitivity response following chicory supplementation<br>– Increased humoral immune response to sheep erythrocytes with chicory supplementation tendency<br>– Altered peripheral CD4+ and CD8+ lymphocyte subpopulations in sorghum-based chicory diet<br>– Higher digestibility of dry matter, organic matter, and total carbohydrates in	[123]

Ingredient	Mode of incorporation/usage	Supplementary form	Inclusion and comparator	Species, sample and design	Reported findings / direction	Ref.
					sorghum-based control diet	
Fiber-prebiotic blend composed of cellulose, beet pulp, inulin, mannan-oligosaccharide, and fructo-oligosaccharide, with or without saccharin–eugenol additive	Fiber-prebiotic blend and saccharin-eugenol additive were incorporated directly into diet formulations before extrusion	Dry extruded dog food	– Control diet: 5% cellulose inclusion<br>– Fiber-prebiotic blend diet: 5% blend containing cellulose, beet pulp, inulin, mannan-oligosaccharides, and fructo-oligosaccharides<br>– Saccharin-eugenol diet: 0.02% saccharin and eugenol additive<br>– Combined diet: 5% fiber-prebiotic blend plus 0.02% saccharin and eugenol<br>– Diets formulated to meet or exceed AAFCO nutrient recommendations for adult dogs	8 intact adult female Beagle dogs; mean age 4.2 ± 1.1 years; mean body weight 10.8 ± 1.4 kg; mean body condition score 5.6 ± 0.6; replicated 4 × 4 Latin square design; four experimental diets including cellulose control, fiber-prebiotic blend, saccharin-eugenol blend, and combined treatment; 14-day experimental periods including 10-day diet adaptation and 4-day fecal and urine collection; dogs individually housed in temperature- and light-controlled rooms	– Increased fecal acetate and propionate concentrations with fiber-prebiotic blend supplementation<br>– Reduced fecal isobutyrate and isovalerate concentrations compared with cellulose control diet<br>– Increased total dietary fiber digestibility in dogs fed combined fiber-prebiotic and saccharin-eugenol diet<br>– Maintained normal fecal quality and stool consistency across treatments<br>– High overall macronutrient digestibility maintained with all dietary treatments	[121]
Xylooligosaccharides as plant xylan-derived prebiotic oligosaccharides	Xylooligosaccharides were incorporated directly into the diet formulation at graded supplementation concentrations before extrusion	Dry extruded cat food	– Control diet: basal diet without xylooligosaccharide supplementation<br>– Low-dose xylooligosaccharide diet: 0.04% supplementation	24 neutered adult domestic shorthair cats (8 males and 16 females); mean age 8.3 ± 2.7 years; mean body weight 3.5 ± 0.8 kg; mean body condition score 4.8 ± 0.8/9; 3-week adaptation period;	– Increased fecal Bifidobacterium populations with xylooligosaccharide supplementation<br>– Increased fecal Lactobacillus populations at selected	[131]

Ingredient	Mode of incorporation/usage	Supplementary form	Inclusion and comparator	Species, sample and design	Reported findings / direction	Ref.
			– Medium-dose xylooligosaccharide diet: 0.07% supplementation<br>– High-dose xylooligosaccharide diet: 0.10% supplementation<br>– Positive-control diet: 0.40% fructooligosaccharide supplementation<br>– All diets formulated to meet AAFCO nutrient recommendations for adult cats at maintenance	cats equally allocated into three dietary groups considering age, sex, and body weight balance; eight cats per group; control versus two xylooligosaccharide-supplemented diets; wheat-free extruded diets containing 36% poultry meal; individually monitored feeding	xylooligosaccharide inclusion levels<br>– Increased fecal acetate and total short-chain fatty acid concentrations<br>– Reduced fecal isobutyrate and isovalerate concentrations compared with control diet<br>– Reduced fecal pH following xylooligosaccharide supplementation<br>– Reduced fecal odor compound concentrations associated with protein fermentation<br>– Maintained normal nutrient digestibility and fecal quality during supplementation	
Malt extract derived from malted barley grains containing soluble carbohydrates and β-glucans	Malt extract was added after extrusion during kibble coating together with swine fat, poultry viscera oil, and liquid palatant	Dry extruded dog food coated with malt extract	– Control diet: 0% malt extract supplementation<br>– Malt diet: 1.0% malt extract inclusion (natural matter basis) applied during coating step after extrusion<br>– Malt extract composition (DM basis): total sugars 73.20%, maltose 34.09%, maltotriose 11.33%,	12 healthy neutered adult dogs; six Beagles and six English Cocker Spaniels; three males and three females of each breed; mean age 2 years; mean body weight 13.45 ± 1.76 kg; mean body condition score 5.25 ± 0.44/9; randomized into two dietary groups (n =	– Increased abundance of Firmicutes after malt extract consumption<br>– Increased *Clostridium* cluster XIVa and *Faecalibacterium* populations<br>– Reduced *Bifidobacterium* abundance<br>– Reduced metabolizable energy of the diet	[124]

Ingredient	Mode of incorporation/usage	Supplementary form	Inclusion and comparator	Species, sample and design	Reported findings / direction	Ref.
			maltotetraose 5.90%, β-glucan-containing malt derivative	6/group); control versus 1.0% malt extract diet; dogs housed in social groups according to affinity; extruded maintenance diets	– Increased lymphocyte proliferation index and proliferation rate<br>– Increased CD4+:CD8+ lymphocyte ratio<br>– Demonstrated prebiotic activity in healthy adult dogs	
Citrus-fruit pectin (Pectin Classic CU201) selected from in vitro screening of soluble fermentable substrates; lactitol used as a non-plant prebiotic comparator	Pectin Classic CU201 or lactitol was administered orally immediately before the first daily meal while dogs consumed the same commercial dry diet throughout the study	Commercial dry dog food with daily oral prebiotic supplementation	– Citrus-fruit pectin (Pectin Classic CU201): 10 g/kg diet for 30 days<br>– Lactitol: 10 g/kg diet for 30 days<br>– Pectin Classic CU201: citrus-derived pectin with degree of esterification between 70% and 74%	16 healthy privately owned adult dogs; different breeds and household environments; mean age 2.3 years (range 1–3 years); mean body weight 21.6 kg (range 10–30 kg); dogs consumed prebiotic- and probiotic-free diets for ≥1 month before enrollment; two treatment groups (n = 8/group); all dogs fed the same commercial dry diet; group 1 received lactitol and group 2 received citrus-fruit pectin at 10 g/kg diet; supplementation for 30 days; fecal samples collected at days 0, 20, 30, and 40	– Reduced fecal coliform counts during supplementation with lactitol or citrus-fruit pectin<br>– Reduced fecal Clostridium perfringens counts over time<br>– In vitro, citrus-fruit pectin increased acetate and butyrate production<br>– In vitro, citrus-fruit pectin reduced ammonia concentration during fermentation<br>– Findings supported selective modulation of fecal bacterial populations and fermentative activity	[149]
1-Kestose as a fructooligosaccharide prebiotic supplement	Kestose powder was orally supplemented once	Oral prebiotic supplementation	– Kestose supplementation: 2 g/day per dog	6 healthy adult Beagle dogs; three males and three females; age range	– Increased fecal Bifidobacterium	[144]

Ingredient	Mode of incorporation/usage	Supplementary form	Inclusion and comparator	Species, sample and design	Reported findings / direction	Ref.
	daily together with the habitual maintenance diet	administered with regular dog food	– Duration of supplementation: 8 weeks<br>– Prebiotic source: kestose-rich fructooligosaccharide preparation	2–6 years; mean body weight 9.9 ± 0.8 kg; individually housed; dogs maintained on the same commercial maintenance diet throughout the study; kestose administered orally once daily for 8 weeks	abundance after kestose supplementation<br>– Increased fecal butyrate concentration after 8 weeks of supplementation<br>– Modulation of canine fecal microbiota toward saccharolytic bacterial populations<br>– Demonstrated bifidogenic activity of kestose in healthy dogs	
Passiflora incarnata extract, *Withania somnifera* extract, and *Taraxacum officinale* root extract containing inulin	Herbal extracts were administered orally once daily as nutraceutical supplementation in addition to the regular commercial diet	Commercial dry dog food with daily oral phytotherapeutic supplementation	– Passiflora incarnata extract: 10 mg/kg body weight/day standardized to 4% vitexin<br>– Withania somnifera extract: 10 mg/kg body weight/day standardized to 2.5% withanolides<br>– Taraxacum officinale root extract: 15 mg/kg body weight/day standardized to 20% inulin<br>– Supplementation duration: 40 days	7 senior dogs (>7 years old); four males and three females; mean body weight 53 ± 2 kg; kennel-housed; individually maintained in indoor–outdoor runs with environmental enrichment; no antibiotic treatment during previous 6 months; dogs continuously fed the same commercial adult dry food throughout the study; oral supplementation for 40 days	– Reduced serum C-reactive protein concentrations after supplementation<br>– Reduced IL-6 concentrations during treatment<br>– Increased total antioxidant capacity in supplemented senior dogs<br>– Reduced serum lipid peroxidation markers<br>– Increased relative abundance of short-chain fatty acid-producing bacterial taxa<br>– Modulation of gut microbiota composition toward potentially beneficial fermentative populations	[132]

Ingredient	Mode of incorporation/usage	Supplementary form	Inclusion and comparator	Species, sample and design	Reported findings / direction	Ref.
					– Supported antioxidant and anti-inflammatory status in aged dogs	

Table S1. 4. Legume-derived protein–carbohydrate matrices

Ingredient	Mode of incorporation/usage	Supplementary form	Inclusion and comparator	Species, sample and design	Reported findings / direction	Ref.
Whole soybean as a legume-based protein–fat–fiber matrix	Whole soybeans replaced portions of corn gluten meal, chicken fat, and brewers rice in the diet formulation before extrusion	Dry extruded dog food	– Four experimental diets: control diet without whole soybeans, 10%, 20% and 30% whole soybeans	12 healthy adult Beagles (8 neutered males and 4 spayed females); mean age 6.25 ± 0.45 years; initial body weight 12.28 ± 1.51 kg; replicated 4 × 4 Latin square design; four dietary treatments (0%, 10%, 20%, or 30% whole soybeans); 9-day dietary adaptation followed by 5-day total fecal collection during each period; total study duration 56 days; individually housed in metabolic pens; diets fed twice daily according to metabolizable energy requirements	– Increased fecal acetate, propionate, and total short-chain fatty acid concentrations with higher whole soybean inclusion<br>– Reduced fecal branched-chain fatty acid concentrations<br>– Lower fresh fecal pH with increasing soybean levels<br>– Increased fecal moisture, output, and defecation frequency<br>– Maintained acceptable stool quality despite higher fiber intake<br>– Greater first-choice preference for soybean-containing diets during palatability testing<br>– Enhanced hindgut fermentative activity associated with soybean-derived fiber and oligosaccharides	[98]
Pulse-based grain-free diet matrices based on red lentil flour and high-amylose wrinkled pea flour, with white rice flour as grain control	Each flour was incorporated into complete experimental diet formulations before extrusion; diets were air-dried for 48 h and	Dry extruded dog food	– Three test diets: white rice flour diet, red lentil flour diet, and wrinkled pea flour diet<br>– All test diets were formulated to contain 15%	8 adult Beagle dogs (4 spayed females, 4 neutered males); age 3–5 years; body weight 9.4 ± 0.4 kg; blinded repeated-measures sequential feeding design;	– Reduced crude protein digestibility with lentil and wrinkled pea diets compared with rice diet	[106]

Ingredient	Mode of incorporation/usage	Supplementary form	Inclusion and comparator	Species, sample and design	Reported findings / direction	Ref.
	vacuum-coated with oil and chicken fat after extrusion		enzymatic starch<br>– wrinkled pea diet required high pulse-flour inclusion, approximately 58%, to achieve 15% enzymatic starch; lentil inclusion was lower, approximately 42%<br>– Total high molecular weight dietary fiber: rice diet 15.6%, lentil diet 16.7%, wrinkled pea diet 17.2% on a dry matter basis<br>– Amylose content: rice diet 4.6%, lentil diet 6.7%, wrinkled pea diet 12.8%	minimum 28-day pre-trial period on commercial grain-containing diet; feeding order: commercial diet, rice diet, lentil diet, rice diet, and wrinkled pea diet; each experimental diet fed continuously for 28 days; rice diet repeated as control and washout; dogs group-housed during daytime and individually kenneled during feeding and overnight; individually measured portions fed twice daily	– Reduced fat digestibility with wrinkled pea diet compared with rice diet<br>– Reduced starch digestibility with wrinkled pea diet compared with rice and lentil diets<br>– Reduced methionine and cystine + methionine levels digestibility with pulse-based diets compared with rice diet<br>– Lower fecal bile acid excretion with wrinkled pea diet<br>– Lower fasting blood glucose with lentil diet<br>– Increased plasma N-Terminal Pro-B-type Natriuretic Peptide (NT-ProBNP) after wrinkled pea diet feeding<br>– Increased left ventricular internal diameter at systole after wrinkled pea diet feeding<br>– Reduced stroke volume after lentil and wrinkled pea diet feeding<br>– Reduced cardiac output after wrinkled pea diet feeding compared with commercial baseline diet	

Ingredient	Mode of incorporation/usage	Supplementary form	Inclusion and comparator	Species, sample and design	Reported findings / direction	Ref.
Dehulled faba beans (*Vicia faba L.*)	Dehulled faba bean flour replaced wheat middlings and corn starch in the diet formulation before extrusion processing	Dry extruded dog food	– Four experimental diets: control diet without dehulled faba beans, 10%, 20%, and 30% dehulled faba beans<br>– All diets formulated to be isonitrogenous and isocaloric and to meet AAFCO nutrient profiles for adult dogs<br>– Experimental diets contained approximately 26% crude protein and 14% crude fat (dry matter basis)	12 healthy adult Beagles (8 neutered males and 4 spayed females); mean age 6.25 ± 0.45 years; initial body weight 12.28 ± 1.51 kg; replicated 4 × 4 Latin square design; four dietary treatments containing 0%, 10%, 20%, or 30% dehulled faba beans; each period consisted of 9-day dietary adaptation followed by 5-day total fecal collection; total study duration 56 days; dogs individually housed in metabolism pens; diets fed twice daily according to metabolizable energy requirements; water available ad libitum	– Increased fecal acetate, propionate, and total short-chain fatty acid concentrations with increasing faba bean inclusion<br>– Reduced fecal branched-chain fatty acid concentrations<br>– Lower fecal pH associated with higher fermentative activity<br>– Increased fecal output and fecal moisture with higher dietary faba bean levels<br>– Maintained acceptable stool quality across treatments<br>– Reduced apparent total tract digestibility of dry matter and organic matter at higher inclusion levels<br>– Increased fermentative substrate delivery to the hindgut from dehulled faba bean fiber fractions	[109]
Red gram (*Cajanus cajan*) and lentil (*Lens culinaris*)	Ground red gram or lentil seeds were incorporated into rice-based homemade diets by replacing 50% of soybean meal protein;	Home-prepared cooked dog diet	– Three diets: soybean meal control diet, red gram diet, and lentil diet<br>– Soybean meal control diet: rice 622 g/kg fresh basis and soybean meal	15 healthy adult Spitz dogs; age approximately 4 years; body weight 4.15–6.95 kg (mean 5.37 ± 0.22 kg); completely randomized design; three	– Maintained normal body weight and general health status throughout feeding period	[110]

Ingredient	Mode of incorporation/usage	Supplementary form	Inclusion and comparator	Species, sample and design	Reported findings / direction	Ref.
	diets were pressure-cooked before feeding		300 g/kg fresh basis<br>– Red gram diet: rice 442 g/kg, soybean meal 160 g/kg, and red gram 320 g/kg fresh basis<br>– Lentil diet: rice 502 g/kg, soybean meal 160 g/kg, and lentil 260 g/kg fresh basis	dietary groups with five dogs per treatment; control soybean meal diet versus partial replacement with red gram or lentil; individually housed in separate kennel cubicles; daily exercise and socialization provided; veterinary-monitored throughout the study; dogs dewormed and vaccinated before trial initiation; feeding trial conducted under institutional animal ethics approval	– Improved crude fiber digestibility in legume-fed groups<br>– Increased antioxidant status with lentil-containing diet<br>– Reduced lipid peroxidation indices in dogs fed legume-based diets<br>– Maintained normal hematological and serum biochemical profiles<br>– Demonstrated suitability of red gram and lentil as alternative plant-derived protein ingredients in canine diets	
Red lentil pasta by-product as an upcycled pulse-based protein–carbohydrate matrix	Red lentil pasta by-product was incorporated into complete extruded dog diets by replacing broken rice as the main carbohydrate source	Dry extruded dog food	– Control diet: broken rice 571.7 g/kg as fed<br>– 33% replacement diet: broken rice 381.2 g/kg and red lentil pasta by-product 190.6 g/kg as fed<br>– 66% replacement diet: broken rice 190.6 g/kg and red lentil pasta by-product 381.2 g/kg as fed<br>– 100% replacement diet: red lentil pasta by-product 571.7 g/kg as fed<br>– A separate substitution diet containing 70%	40 healthy adult Beagle dogs (19 males and 21 females, all neutered); median age 3 years (interquartile range 2–7 years); body weight 11.9 ± 2.0 kg; median body condition score 5/9; randomly assigned to five dietary groups; control rice-based diet versus diets containing 33%, 66%, or 100% rice replacement with red lentil pasta by-product	– Increased fecal acetate, propionate, total short-chain fatty acids, and lactate concentrations with increasing lentil pasta by-product inclusion<br>– Reduced fecal pH with higher lentil pasta by-product levels<br>– Increased fecal moisture and fecal output with graded lentil inclusion<br>– Maintained fecal scores despite increased fermentative activity	[107]

Ingredient	Mode of incorporation/usage	Supplementary form	Inclusion and comparator	Species, sample and design	Reported findings / direction	Ref.
			control diet plus 30% red lentil pasta by-product was used to estimate the digestibility of the ingredient itself<br>– All diets formulated to meet FEDIAF nutrient requirements for adult dogs		– Lower postprandial glucose and insulin responses in dogs fed the 100% lentil pasta by-product diet<br>– Increased fecal spermidine concentration associated with microbial fiber fermentation<br>– Improved first-choice preference and consumption rate for the 100% rice replacement diet during palatability testing<br>– Maintained nutrient digestibility at 33% and 66% rice replacement levels	
Thermally treated chickpea flour or pea flour in complementary vegetable foods	Pulses were autoclave-treated, hot-air dried, and milled before incorporation. Chickpea flour or pea flour was incorporated into complementary food formulations and mixed daily with raw beef meat before feeding	Raw meat-based dog diet complemented with pulse-containing vegetable food	– Chickpea diet (CP): complementary food containing 15% chickpea flour mixed with 70% raw beef meat<br>– Pea diet (PE): complementary food containing 15% pea flour mixed with 70% raw beef meat<br>– Complementary foods also contained rice flour, oat flakes, dried ground carrots, algae-derived	8 healthy adult Border Collie dogs (4 males and 4 females; mean body weight 15.3 ± 2.0 kg; body condition score 4/9); crossover design; dogs initially fed a commercial extruded reference diet, then divided into two groups receiving either chickpea- or pea-based diets for 14 days followed by diet inversion for another 14 days; diets consisted of raw beef meat	– Altered fecal microbiome composition following substitution of commercial diet with pulse-containing raw meat diets<br>– Increased relative abundance of *Erysipelotrichaceae incertae sedis*, *Eubacterium*, Anaerobacter, and Sarcina in pulse-fed dogs compared with reference diet<br>– Differential modulation of *Prevotella*, *Lactobacillus*,	[111]

Ingredient	Mode of incorporation/usage	Supplementary form	Inclusion and comparator	Species, sample and design	Reported findings / direction	Ref.
			omega-3 fatty acids, and vitamin-mineral premix<br>– Reference diet (RD): commercial extruded complete dog food	combined with complementary foods containing chickpea or pea flour; individually housed in private kennel; daily fresh diet preparation	*Alloprevotella*, and *Sutterella* between chickpea- and pea-based diets<br>– Increased fecal isovalerate concentration in both chickpea and pea diets relative to reference diet<br>– Maintained fecal quality and fermentation metabolite profiles despite dietary transition<br>– Demonstrated diet-dependent modulation of canine fecal microbial ecology by pulse type	
Legume-based novel protein–carbohydrate matrices: garbanzo bean, green lentil, and peanut flour, with dried yeast and poultry by-product meal control	Each protein source was incorporated into the diet formulation before extrusion as the primary dietary protein ingredient replacing conventional animal-protein components	Dry extruded dog food	– Five diets formulated with either poultry by-product meal control, garbanzo beans, green lentils, peanut flour, or dried yeast as the primary protein source<br>– Diet crude protein ranged from 26.6% to 31.2% dry matter basis<br>– Total dietary fibre ranged from 10.5% to 17.7% dry matter basis<br>– Dried yeast diet contained the highest total dietary fibre concentration (17.7% dry matter basis)	10 adult intact female Beagle dogs; mean age 5.0 ± 1.2 years; mean body weight 11.9 ± 1.3 kg; mean body condition score 5.9/9; replicated 5 × 5 Latin square design; five dietary treatments consisting of poultry by-product meal control, garbanzo bean, green lentil, peanut flour, or dried yeast diets; 14-day experimental periods with 10-day diet adaptation	– Legume- and yeast-based diets maintained high macronutrient apparent total tract digestibility (>80%) without detrimental gastrointestinal effects<br>– Plant-based protein diets increased total fecal short-chain fatty acid concentrations compared with poultry by-product meal control<br>– Dried yeast diet markedly increased fecal butyrate concentration	[112]

Ingredient	Mode of incorporation/usage	Supplementary form	Inclusion and comparator	Species, sample and design	Reported findings / direction	Ref.
			– All diets were formulated to meet AAFCO nutrient recommendations for adult dogs at maintenance		compared with all other diets<br>– Garbanzo bean diet increased fecal acetate and propionate concentrations<br>– Green lentil and peanut flour diets reduced fecal phenol and indole concentrations compared with control<br>– Fecal pH was reduced in dogs fed garbanzo bean, green lentil, and dried yeast diets<br>– Dried yeast diet produced distinct fecal microbial β-diversity compared with all other dietary treatments<br>– Garbanzo bean and green lentil diets increased relative abundance of *Megamonas*, a genus associated with oligosaccharide fermentation	
Pulse-based carbohydrate matrices from lentil, smooth pea, fava bean, and high-amylose wrinkled pea flours, with white rice flour as grain control	Rice or pulse flours were incorporated into complete canine diet formulations as the principal available-carbohydrate source before extrusion; kibble was	Dry extruded dog food	– Six test diets: rice flour, red lentil flour, smooth pea flour, fava bean flour, wrinkled pea, and wrinkled pea Amigold flour	8 healthy adult Beagle dogs; four spayed females and four neutered males; 2–4 years old; mean body weight 8.87 ± 0.90 kg; randomized, crossover, blinded, repeated-	– Reduced fasting and post-prandial blood glucose responses with lentil and wrinkled pea diets	[108]

Ingredient	Mode of incorporation/usage	Supplementary form	Inclusion and comparator	Species, sample and design	Reported findings / direction	Ref.
	subsequently dried and fat-coated		– Diets were formulated to provide approximately 20% available carbohydrate<br>– Flour inclusion, as fed: rice 23.12%, lentil 42.19%, smooth pea 41.67%, fava bean 46.40%, wrinkled pea 4,140–4 58.65%, and wrinkled pea Amigold 58.14%	measures feeding design; six dietary treatments including rice, lentil, smooth pea, fava bean, wrinkled pea 4,140–4, and wrinkled pea Amigold diets; each diet fed for 7 consecutive days; 3-day washout period with commercial diet between treatments; complete dry diets coated with chicken fat and canola oil before feeding	– Lower glycemic index relative to rice-based control diet<br>– Reduced apparent starch digestibility in high-amylose wrinkled pea diets<br>– Altered digestibility of sulfur-containing amino acids and taurine among pulse-based diets<br>– Reduced fecal bile acid excretion with higher dietary fiber and amylose content<br>– Maintenance of plasma taurine concentrations within physiological reference ranges after short-term pulse feeding	

Table S1. 5. Fermented plant-derived ingredients

Ingredient	Mode of incorporation/usage	Supplementary form	Inclusion and comparator	Species, sample and design	Reported findings / direction	Ref.
Fermented rye and unfermented rye cereal matrices, compared with corn meal, in a vegetarian base diet co-supplemented with hydrolyzed feather meal	Hydrolyzed feather meal and either corn meal, fermented rye, or rye were manually mixed daily into the vegetarian basal diet before feeding; fermented rye was produced by controlled liquid fermentation using lactic acid bacterial starter cultures	Semi-moist dog diet	– Vegetarian basal diet composed of wheat, broken rice, wheat gluten, rice protein, linseed, sunflower oil, and dried beet pulp<br>– Feather meal supplementation: 2.7% as-is basis in all non-vegetarian diets<br>– Corn meal diet: 20.1% corn meal as-is basis<br>– Rye diet: 20.1% rye as-is basis<br>– Fermented rye diet: 60.4% fermented rye as-is basis (fermented rye DM: 30.7%)<br>– Approximately 40% added water in non-fermented diets to standardize consistency	8 healthy adult female Beagle dogs; mean BW 11.0 ± 1.31 kg; median age 3 years (28–76 months); 4 × 4 Latin square design; vegetarian basal diet vs feather-meal diets containing corn meal, fermented rye, or rye; each experimental period lasted 10 days with 5-day adaptation and fecal collection phase; 4-day washout between periods; individually housed during digestibility testing with social contact maintained; diets fed once daily according to energy requirements	– Comparable fecal quality and stool consistency across rye-containing and control diets<br>– High acceptance and complete consumption of all experimental diets<br>– High overall nutrient digestibility maintained with rye inclusion<br>– Lower starch digestibility observed in the corn meal diet relative to other diets<br>– Fermented rye supported acceptable fecal moisture and consistency under high-moisture feeding conditions	[114]
Freeze-dried fermented soybean product (natto) containing *Bacillus subtilis* var. *natto* spores, poly-γ-glutamic acid, levan, nattokinase, polyamines, and vitamin K2	Freeze-dried natto product was orally administered once daily together with the regular commercial dry diet	Commercial dry dog food with daily natto supplementation	– Natto-treated group: 10 g freeze-dried natto product/dog/day for 14 days<br>– Untreated group: regular commercial dry diet only<br>– Regular diet provided at 20 g/kg BW/day<br>– Natto product contained approximately 100 billion	8 healthy adult dogs (six miniature poodles and two mixed-breed dogs); mean age 2.38 ± 0.73 years; mean BW 5.88 ± 1.32 kg; randomized into natto-treated (n = 4) and untreated control (n = 4) groups; individually housed and regularly	– Increased peripheral natural killer (NK) cell populations<br>– Enhanced PBMC cytotoxic activity against canine tumor cells<br>– Increased TNF-α expression following antigen stimulation	[126]

Ingredient	Mode of incorporation/usage	Supplementary form	Inclusion and comparator	Species, sample and design	Reported findings / direction	Ref.
			B. subtilis natto organisms/100 g	exercised; commercial dry diet feeding with or without freeze-dried natto supplementation; 14-day feeding period	– Augmented cellular immune activity associated with fermented soybean supplementation	
Aspergillus oryzae fermented soybean meal, soybean meal with *A. oryzae* biomass product, and soybean protein isolate	Fermented soybean meal or soybean protein ingredients were incorporated into complete diet formulations before extrusion; chicken fat and dry digest flavor were coated onto kibble after drying	Dry extruded dog food	– Fermented soybean meal was produced by inoculating soybean meal with *Aspergillus oryzae* at 1 × 10^4 spores/g soybean meal dry matter<br>– Fermentation conditions: 37°C, 80% relative humidity, 36 h fermentation, followed by oven-drying at 60°C for 36 h and grinding through a 1.00 mm sieve<br>– Four diets: 30% soybean meal diet, 30% soybean meal + 1% dried *Aspergillus oryzae* biomass product diet, 30% fermented soybean meal diet, and 18% soybean protein isolate diet<br>– All diets contained 0.4% titanium dioxide as an indigestible marker	12 healthy adult Beagles (6 neutered males and 6 spayed females); mean age 3.0 ± 0.2 years; mean BW 9.8 ± 1.6 kg; replicated 4 × 4 Latin square design; individually housed in metabolic pens; four experimental periods with 9-day diet adaptation and 5-day fecal collection; dogs fed twice daily to maintenance energy requirements	– Improved dry matter digestibility with fermented soybean meal relative to conventional soybean meal<br>– Increased fecal acetate, propionate, and total SCFAs in dogs fed diets containing soybean meal and A. oryzae biomass product<br>– Lower fecal fermentation metabolite production in soybean protein isolate diet<br>– Maintained acceptable stool quality and fecal consistency across diets<br>– Reduced palatability observed for fermented soybean meal relative to conventional soybean meal	[115]
Fermented plant protein (Proteger®), an enzyme-enhanced fungal	Fermented plant protein replaced soybean meal at graded inclusion levels in complete diet formulations	Dry extruded dog food	– Control diet (Soybean meal): 15% soybean meal without fermented plant protein	12 healthy adult Beagle dogs; replicated 4 × 4 Latin square design; four experimental diets with	– Increased crude protein digestibility at the highest fermented plant protein inclusion level	[116]

Ingredient	Mode of incorporation/usage	Supplementary form	Inclusion and comparator	Species, sample and design	Reported findings / direction	Ref.
fermented soybean meal product	before extrusion; diets were processed by single-screw extrusion and coated with chicken fat and dry digest		– 5% fermented plant protein replacing soybean meal<br>– 10% fermented plant protein replacing soybean meal<br>– 15% fermented plant protein replacing soybean meal<br>– All diets contained 0.4% titanium dioxide as digestibility marker	graded fermented plant protein inclusion; 9-day adaptation followed by 5-day total fecal collection per period; individually housed during collection phase; fresh fecal samples collected for hindgut fermentation analysis; nutrient digestibility determined by total fecal collection and titanium dioxide marker methods; two-bowl palatability testing performed separately	– Maintained acceptable fecal quality and stool consistency across diets<br>– Maintained fecal fermentation profile without adverse shifts in fecal pH or ammonia<br>– Increased kibble sectional expansion during extrusion processing<br>– Maintained overall food intake and diet acceptance despite graded fermented protein inclusion	
Fermented plant protein (Proteger®), a fungal-fermented soybean protein ingredient	Fermented plant protein replaced soybean meal at graded levels in complete feline diet formulations before extrusion; diets were coated with chicken fat and flavor digest after drying	Dry extruded cat food	– Control diet (Soybean meal): 15% soybean meal without fermented plant protein<br>– 5% fermented plant protein replacing soybean meal<br>– 10% fermented plant protein replacing soybean meal<br>– 15% fermented plant protein replacing soybean meal<br>– All diets contained 0.4% titanium dioxide as digestibility marker	Twelve healthy intact male domestic shorthair cats; mean age 0.8 ± 0.1 years; mean BW 4.8 ± 0.6 kg; replicated 4 × 4 Latin square design; four dietary treatments with graded fermented plant protein inclusion; each experimental period included 10-day adaptation followed by 4-day total fecal collection; individually housed during collection phase; diets fed twice daily to maintain body weight	– Maintained acceptable fecal quality and stool consistency across all fermented plant protein inclusion levels<br>– Improved crude protein digestibility with increasing fermented plant protein inclusion<br>– Reduced fecal phenol and indole concentrations with fermented plant protein diets<br>– Maintained diet acceptance and food intake across treatments	[113]

Ingredient	Mode of incorporation/usage	Supplementary form	Inclusion and comparator	Species, sample and design	Reported findings / direction	Ref.
					– Improved extrusion processing characteristics and kibble expansion with fermented plant protein inclusion	
Candida utilis–fermented pea starch compared with unfermented pea starch	Fermented pea starch, unfermented pea starch, or corn starch were incorporated into complete diet formulations before extrusion as the principal starch source	Dry extruded dog and cat food	– Whole diets contained approximately 30% starch inclusion from either *C. utilis*-fermented pea starch, unfermented pea starch, or corn starch control<br>– Fermented pea starch produced through solid-state fermentation using *Candida utilis*<br>– Separate but compositionally matched diets formulated for dogs and cats	Adult dogs and cats evaluated in separate feeding trials; randomized dietary allocation to control corn starch, unfermented pea starch, or *C. utilis*-fermented pea starch diets; digestibility, blood metabolites, fecal characteristics, microbiota, and intestinal health markers assessed after dietary adaptation; complete extruded diets fed to maintain body weight and normal health status	– Reduced post-prandial glycemic response with fermented pea starch diets<br>– Increased fecal short-chain fatty acid production<br>– Beneficial modulation of fecal microbial populations<br>– Improved markers of intestinal and metabolic health<br>– Maintained acceptable nutrient digestibility and stool quality across diets	[27]
Candida utilis-fermented pea starch compared with unfermented pea starch and corn starch	Fermented pea starch, unfermented pea starch, or corn starch were incorporated into complete diet formulations before extrusion as the principal carbohydrate source	Dry extruded dog and cat food	– Whole diets contained approximately 30% starch from either *C. utilis*-fermented pea starch, unfermented pea starch, or corn starch control<br>– Fermented pea starch produced through solid-state fermentation using *Candida utilis*	Healthy adult dogs and cats evaluated in separate randomized feeding trials; control corn starch, unfermented pea starch, and *C. utilis*-fermented pea starch diets compared; feeding periods included dietary adaptation and sample collection phases; complete extruded diets	– Reduced post-prandial glycemic response<br>– Increased fecal SCFA production<br>– Enhanced saccharolytic fermentation activity<br>– Beneficial modulation of fecal microbial ecology<br>– Improved indicators of intestinal and metabolic health	[142]

Ingredient	Mode of incorporation/usage	Supplementary form	Inclusion and comparator	Species, sample and design	Reported findings / direction	Ref.
			– Experimental diets formulated to be nutritionally comparable within species	fed to maintain body weight		
Candida utilis-fermented low- or high-tannin fava bean flour relative to unfermented fava bean flour, with commercial normal- and high-protein diets as comparators	– Fermentation organism: *Candida utilis* ATCC 9950<br>– Fermentation processing: 20 kg fava bean flour batches blended with yeast broth, ammonia, and sterile water; fermented at 30°C for 72 h; mixed for 3 min every hour; dried at 60°C for 48 h before diet manufacture<br>– Fermented or unfermented low- and high-tannin fava bean flours were incorporated into complete diet formulations before extrusion as pulse-based protein and carbohydrate sources	Dry extruded dog food	– Four fava bean diets: unfermented low-tannin fava bean diet, fermented low-tannin fava bean diet, unfermented high-tannin fava bean diet, and fermented high-tannin fava bean diet<br>– Fava bean flour inclusion: 30% in each fava bean-based diet	8 healthy mixed-gender neutered Beagle dogs; repeated-measures feeding study; commercial normal- and high-protein diets fed during separate feeding periods; randomized crossover 2 × 2 Latin square design used for low- and high-tannin fava bean diets with or without fermentation; each diet fed for 28 days	– Improved glucose tolerance with fermented fava bean diets<br>– Increased red blood cell counts following fermentation<br>– Reduced post-challenge glucose response compared with unfermented diets<br>– Maintained body weight and body condition during pulse-based feeding<br>– No evidence of adverse cardiac effects during 28-day feeding period	[127]
Corn fermented protein compared with distillers dried grains with solubles and brewer's dried yeast	Corn fermented protein, distillers dried grains with solubles, or brewer's dried yeast were incorporated into complete diet formulations before extrusion as alternative fermented corn co-	Dry extruded dog food	– Control diet: 15% soybean meal<br>– Brewer's dried yeast diet: 3.5% brewer's dried yeast<br>– Brewer's dried yeast plus distillers dried grains with solubles diet: 2.5% brewer's dried yeast +	12 healthy adult Beagle dogs; eight castrated males and four spayed females; mean age 6.3 ± 0.45 years; mean body weight 11.4 ± 1.2 kg; triplicated 4 × 4 Latin square design; four experimental periods; 9-day dietary adaptation	– Improved stool consistency with corn fermented protein compared with distillers dried grains with solubles<br>– Higher apparent nutrient digestibility than distillers dried grains with solubles diet	[125]

Ingredient	Mode of incorporation/usage	Supplementary form	Inclusion and comparator	Species, sample and design	Reported findings / direction	Ref.
	products and yeast-derived protein sources		17.5% distillers dried grains with solubles<br>– Corn fermented protein diet: 17.5% corn fermented protein<br>– Titanium dioxide added at 0.40% as digestibility marker	followed by 5-day total fecal collection per period; individually housed in temperature-controlled kennels; twice-daily feeding	– Maintained fecal short-chain fatty acid profiles<br>– Reduced fecal output relative to distillers dried grains with solubles diet<br>– Acceptable palatability in adult dogs	
Corn-fermented protein as a fermented corn co-product protein/fiber ingredient replacing soybean meal	Corn fermented protein was incorporated into complete feline diet formulations before extrusion as a replacement for traditional protein ingredients	Dry extruded cat food	– Control diet: 0% corn fermented protein<br>– Low inclusion diet: 5% corn fermented protein<br>– Moderate inclusion diet: 10% corn fermented protein<br>– High inclusion diet: 15% corn fermented protein<br>– Titanium dioxide included as an indigestible marker for digestibility measurements	11 healthy adult American shorthair cats (10 males and 1 female); mean age 3.1 ± 1.7 years; mean body weight 5.6 ± 1.7 kg; incomplete replicated Latin square design; four experimental periods; 9-day dietary adaptation followed by 5-day collection period; each cat served as its own control; group-housed during adaptation and individually housed during collection; twice-daily feeding	– Maintained acceptable stool quality across graded inclusion levels<br>– Improved apparent crude protein digestibility at selected inclusion levels<br>– Increased fecal short-chain fatty acid production<br>– Maintained fecal pH within normal range<br>– Supported acceptable diet palatability in adult cats	[117]
